# Stabilization of E-cadherin adhesions by COX-2/GSK3**β** signaling is a targetable pathway in metastatic breast cancer

**DOI:** 10.1172/jci.insight.156057

**Published:** 2023-03-22

**Authors:** Kuppusamy Balamurugan, Dipak K. Poria, Saadiya W. Sehareen, Savitri Krishnamurthy, Wei Tang, Lois McKennett, Veena Padmanaban, Kelli Czarra, Andrew J. Ewald, Naoto T. Ueno, Stefan Ambs, Shikha Sharan, Esta Sterneck

**Affiliations:** 1 Laboratory of Cell and Developmental Signaling, Center for Cancer Research (CCR), National Cancer Institute (NCI), Frederick, Maryland, USA.; 2 Morgan Welch Inflammatory Breast Cancer Research Program and Clinic, The University of Texas MD Anderson Cancer Center, Houston, Texas, USA.; 3 Laboratory of Human Carcinogenesis, CCR, NCI, Bethesda, Maryland, USA.; 4 Laboratory Animal Sciences Program, Leidos Biomedical Research Inc., Frederick National Laboratory for Cancer Research, Frederick, Maryland, USA.; 5 Departments of Cell Biology and Oncology, Sidney Kimmel Comprehensive Cancer Center, Johns Hopkins University School of Medicine, Baltimore, Maryland, USA.

**Keywords:** Cell Biology, Oncology, Breast cancer, Cell migration/adhesion, Signal transduction

## Abstract

Metastatic progression of epithelial cancers can be associated with epithelial-mesenchymal transition (EMT) including transcriptional inhibition of E-cadherin (*CDH1*) expression. Recently, EM plasticity (EMP) and E-cadherin–mediated, cluster-based metastasis and treatment resistance have become more appreciated. However, the mechanisms that maintain E-cadherin expression in this context are less understood. Through studies of inflammatory breast cancer (IBC) and a 3D tumor cell “emboli” culture paradigm, we discovered that cyclooxygenase 2 (COX-2; *PTGS2*), a target gene of C/EBPδ (*CEBPD*), or its metabolite prostaglandin E2 (PGE2) promotes protein stability of E-cadherin, β-catenin, and p120 catenin through inhibition of GSK3β. The COX-2 inhibitor celecoxib downregulated E-cadherin complex proteins and caused cell death. Coexpression of E-cadherin and COX-2 was seen in breast cancer tissues from patients with poor outcome and, along with inhibitory GSK3β phosphorylation, in patient-derived xenografts (PDX) including triple negative breast cancer (TNBC).Celecoxib alone decreased E-cadherin protein expression within xenograft tumors, though CDH1 mRNA levels increased, and reduced circulating tumor cell (CTC) clusters. In combination with paclitaxel, celecoxib attenuated or regressed lung metastases. This study has uncovered a mechanism by which metastatic breast cancer cells can maintain E-cadherin–mediated cell-to-cell adhesions and cell survival, suggesting that some patients with COX-2^+^/E-cadherin^+^ breast cancer may benefit from targeting of the PGE2 signaling pathway.

## Graphical abstract

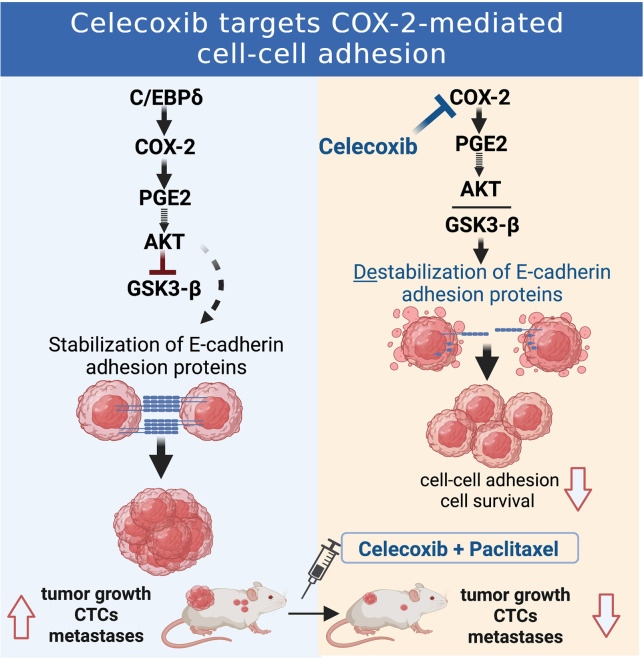

## Introduction

Breast cancer (BC) subtypes are classified by expression of hormone receptors (HR) for estrogen and progesterone as well as HER2. Inflammatory BC (IBC) is a rare, highly invasive subtype of BC that can include any of the classical subtypes but does not have IBC-specific treatment options (1). While the term “inflammatory” has been considered a misnomer for IBC, proinflammatory factors and signaling pathways including prostaglandin-endoperoxide synthase or cyclooxygenase 2 (COX-2) are indeed upregulated in IBC (1–3). Inflammation has been associated with induction of epithelial-mesenchymal transition (EMT) of BC cells, which involves downregulation of E-cadherin gene (*CDH1*) expression and promotes invasiveness (4). However, E-cadherin expression is maintained in many advanced breast cancers including IBC, where it plays an important role in the formation of tumor cell emboli. These clusters of tumor cells within the cancer parenchyma and dermal lymphatic vasculature predict poor outcome (5–7). In addition, it is becoming more evident that cancer cell dissemination may not require complete EMT but rather fluid transitions between EM phenotypes or hybrid states, termed EM plasticity (EMP) (8, 9, 10). Thus, it has been shown that E-cadherin expression can contribute to collective cell migration, establishment of metastases, chemotherapy resistance, and cancer cell survival under hypoxia (8, 11–15). Furthermore, a recent report confirmed that not only is E-cadherin expressed on cluster circulating tumor cells (CTCs), which are highly metastatic and predict poor outcome (16, 17), but is even more abundant than EpCAM, the traditional epithelial marker for CTCs (18). Indeed, among BC subtypes, only lobular carcinoma is marked by downregulation of E-cadherin, while most ductal carcinomas and metastases maintain E-cadherin expression (8, 19) and analysis of large genomic data sets showed a positive correlation of *CDH1* (E-cadherin) gene expression with poor outcome for patients (20). While inhibition of *CDH1* gene expression by EMT transcription factors is a well-described aspect of EMT, such factors can coexist with E-cadherin (21, 22). However, the mechanisms that maintain E-cadherin expression in metastatic BC cells are poorly understood. A detailed understanding of the molecular pathways that foster cell-to-cell adhesion and, thereby, metastatic cancer cell survival will provide new mechanistic insights into BC progression. Experiments with 3D cell culture conditions that were designed to mimic the lymphatic environment showed that IBC cell lines form emboli-like structures in vitro, which resemble emboli in patients (23, 24). This assay system can, thus, be used to interrogate the pathways leading to E-cadherin–mediated cancer cell-to-cell adhesion (25).

We began this study after observing that the transcription factor C/EBPδ (*CEBPD*) was highly expressed in IBC cell lines and in parenchymal tumor cell emboli of patient tissues. In many cell types, C/EBPδ expression is induced by cytokines via STAT3 and NF-κB signaling and participates in the further induction of proinflammatory genes including IL-6 and the IL-6 receptor (26, 27). Within non-IBC, high C/EBPδ protein expression is mostly seen in low-grade, HR^+^ luminal-epithelial tumors, and attenuates cell proliferation, motility, and invasion in HR^+^ cell lines in culture (28). However, in the context of inflammation and hypoxia, C/EBPδ promotes cancer stem cell–associated phenotypes (27). Thus, the role of C/EBPδ depends in part on cell type and context (26). In this report, we show how studies in 3D culture revealed that C/EBPδ supports E-cadherin expression and cell-to-cell adhesions through expression of COX-2, which sets in motion a signaling cascade that leads to stabilization of epithelial cadherin/catenin proteins. We further provide in vivo evidence that the COX-2/E-cadherin pathway extends beyond IBC, may contribute to poor prognosis in BC, and offers potential for targeted therapy.

## Results

### C/EBPδ is expressed in IBC cells and promotes expression of E-cadherin and cell-to-cell adhesion in 3D.

**Figure 1:**
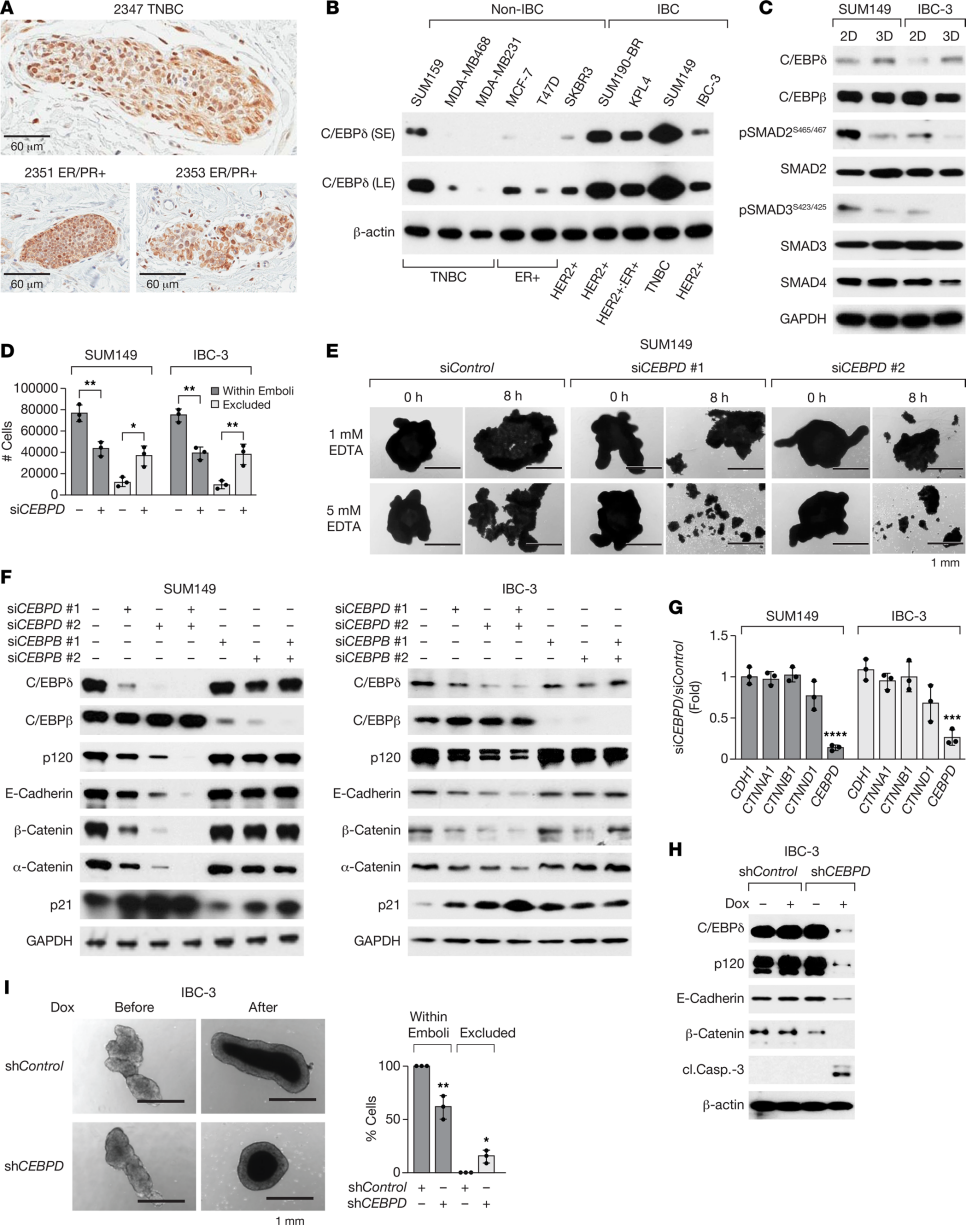
C/EBPδ is expressed in IBC emboli in vivo and IBC cell lines in vitro and promotes cell-to-cell adhesion and E-cadherin protein expression. (**A**) C/EBPδ immunostaining in emboli from 3 IBC patient tissues. Scale bars: 60 μm. (**B**) Western blot analysis of C/EBPδ expression in whole cell extracts of the indicated cell lines and BC subtypes. S/LE, short/long exposure. (**C**) Western blot analysis of indicated proteins in SUM149 and IBC-3 cell lines that were cultured on plastic (2D) or as emboli (3D) for 4 days. (**D**) Quantification of SUM149 or IBC-3 cells, transfected with si*Control* (–) or si*CEBPD* (+) oligos, that aggregated into large clusters (“within emboli”) or remained as single cells/smaller clusters (“excluded”) after 3 days in 3D culture (*n* = 3, mean ± SEM; *P < 0.05, **P < 0.01 compared with si*Control*). (**E**) Images of similarly sized emboli from SUM149 cells, transfected with control or 2 independent si*CEBPD* oligos, before and after treatment with EDTA for 8 hours (representative of 3 experiments). (**F**) Western blot analysis of the indicated proteins in established emboli of SUM149 and IBC-3 cells that had been transfected with siRNAs as indicated. (**G**) qPCR analysis of *CDH1* (E-cadherin), *CTNNA1* (α-catenin), *CTNNB1* (β-catenin), *CTNND1* (p120), and *CEBPD* mRNA levels in emboli of SUM149 and IBC-3 cells transfected with si*CEBPD* relative to si*Control*-transfected (*n* = 3, mean ± SEM; ***P < 0.001, ****P < 0.0001 compared with si*Control*). (**H**) Western blot analysis of IBC-3 cells with stable expression of the indicated inducible shRNA and after culture in 3D for 3 days plus 3 days in the presence of doxycycline (Dox, 100 ng/mL; cl.Casp.-3, cleaved caspase-3). (**I**) Left: Images of representative emboli as in **H** and the same embolus before and after treatment with Dox (10 ng/mL) for 7 days. Scale bar: 1 mm. Right: Quantification of the number of cells in emboli after treatment normalized to untreated control as 100% (*n* = 3, mean ± SEM; *P < 0.05; **P < 0.01).

Because of the implication of inflammation-related signaling pathways in IBC and C/EBPδ’s role in proinflammatory signaling (1, 26, 29), we analyzed C/EBPδ expression in IBC tissues by IHC. Analysis of 39 specimens representing different BC subtypes yielded variable C/EBPδ expression patterns and no significant nuclear staining in most tumor cells. However, in 13 of 14 specimens that also contained tumor cell emboli, nuclear C/EBPδ expression was detectable in cells within emboli (Figure 1A). Our prior analysis of patient-derived xenografts (PDXs) showed that IHC with this antibody for C/EBPδ is specific (28) but not very sensitive (27). Thus, while C/EBPδ expression in IBC overall remained unclear, the results indicate that C/EBPδ can be expressed in cells within emboli that have intravasated into the lymphovascular space. Analysis of BC cell lines, however, revealed that C/EBPδ expression was higher in IBC than most of the non-IBC cell lines tested (Figure 1B). In concordance with our previous studies in non-IBC triple-negative BC (TNBC) (27), C/EBPδ supported in vitro invasiveness, expression of prooncogenic factors (CXCR4, STAT3 and Notch pathway activation), and cancer stem cell markers (CD44^+^/CD24^–^) in SUM149 and IBC-3 cell lines, and it supported growth of established SUM149 experimental metastases in vivo (Supplemental Figure 1, A–G; supplemental material available online with this article; https://doi.org/10.1172/jci.insight.156057DS1). Because C/EBPδ expression in patient tissues was most pronounced in tumor cell emboli, we next employed a 3D in vitro culture model in which cells are seeded in suspension with PEG8000-supplemented media and rocked at slow speed. This paradigm was developed to mimic the mechanophysical environment encountered by the cancer cells within lymphatic vessels (23). Under these conditions (from here on referred to as “3D”), 3 IBC cell lines aggregate into large, tight clusters (from here on referred to as “emboli”), but the 4 tested non-IBC cell lines did not (23). While these clusters “closely resemble IBC patient emboli with respect to size, composition, and E-cadherin expression” (23), we have employed this culture system primarily to model the 3D architecture of tumor cell assemblies in vivo. Consistent with the previously reported abrogated TGF-β signaling pathway in IBC tumors and even more so in emboli (30, 31), culture of SUM149 and IBC-3 cells in 3D reduced the phosphorylation of SMAD2 and SMAD3 compared with adherent cells (2D) grown on plastic dishes (Figure 1C). In addition, IBC-3 cells exhibit reduced levels of the cofactor SMAD4. In contrast, culture in 3D induced *CEBPD* mRNA (Supplemental Figure 1H) and protein expression in both cell lines, though more significantly in IBC-3, which exhibit lower basal levels (Figure 1C). Expression of the related protein C/EBPβ was not induced (Figure 1C). To test whether C/EBPδ plays any role in the emboli formation, C/EBPδ was silenced in SUM149 and IBC-3 cells prior to 3D culture, which resulted in fewer and/or smaller emboli as significantly fewer cells aggregated in 3D (Figure 1D). Studies have shown that tumor cell emboli depend at least in part on cell-to-cell adhesions through E-cadherin, which are dependent on binding of Ca^++^ (6). Thus, we assessed the effect of Ca^++^ chelation by EDTA and found that emboli formed by C/EBPδ-depleted cells dissociated more readily when incubated with EDTA (Figure 1E and Supplemental Figure 1I). Western blot analysis revealed that emboli of cells with knockdown of *CEBPD,* but not of *CEBPB,* contained significantly lower levels of not only E-cadherin protein but also α-catenin, β-catenin, and p120 (Figure 1F), which are part of the E-cadherin adhesion complex (6). However, C/EBPδ depletion did not affect the mRNA levels of the corresponding genes (Figure 1G). Next, we asked if this pathway was only necessary for the process of emboli formation or also for the maintenance of established cell-to-cell adhesions. Doxycycline (Dox) treatment of established IBC-3 emboli downregulated E-cadherin, β-catenin, and p120 proteins and triggered caspase 3 cleavage indicative of cell death in cells when shRNA targeting *CEBPD* was induced (Figure 1H). Accordingly, the number of cells within emboli diminished and cells were also shed from *CEBPD*-depleted emboli (Figure 1I). Before shRNA induction, emboli of both stable lines contained similar numbers of cells (Supplemental Figure 1J). Taken together, these data show that C/EBPδ supports malignant phenotypes in IBC cells as well as the expression of E-cadherin complex proteins and cell-to-cell adhesion.

### C/EBPδ promotes expression of E-cadherin complex proteins through COX-2–mediated GSK3β inhibition.

**Figure 2:**
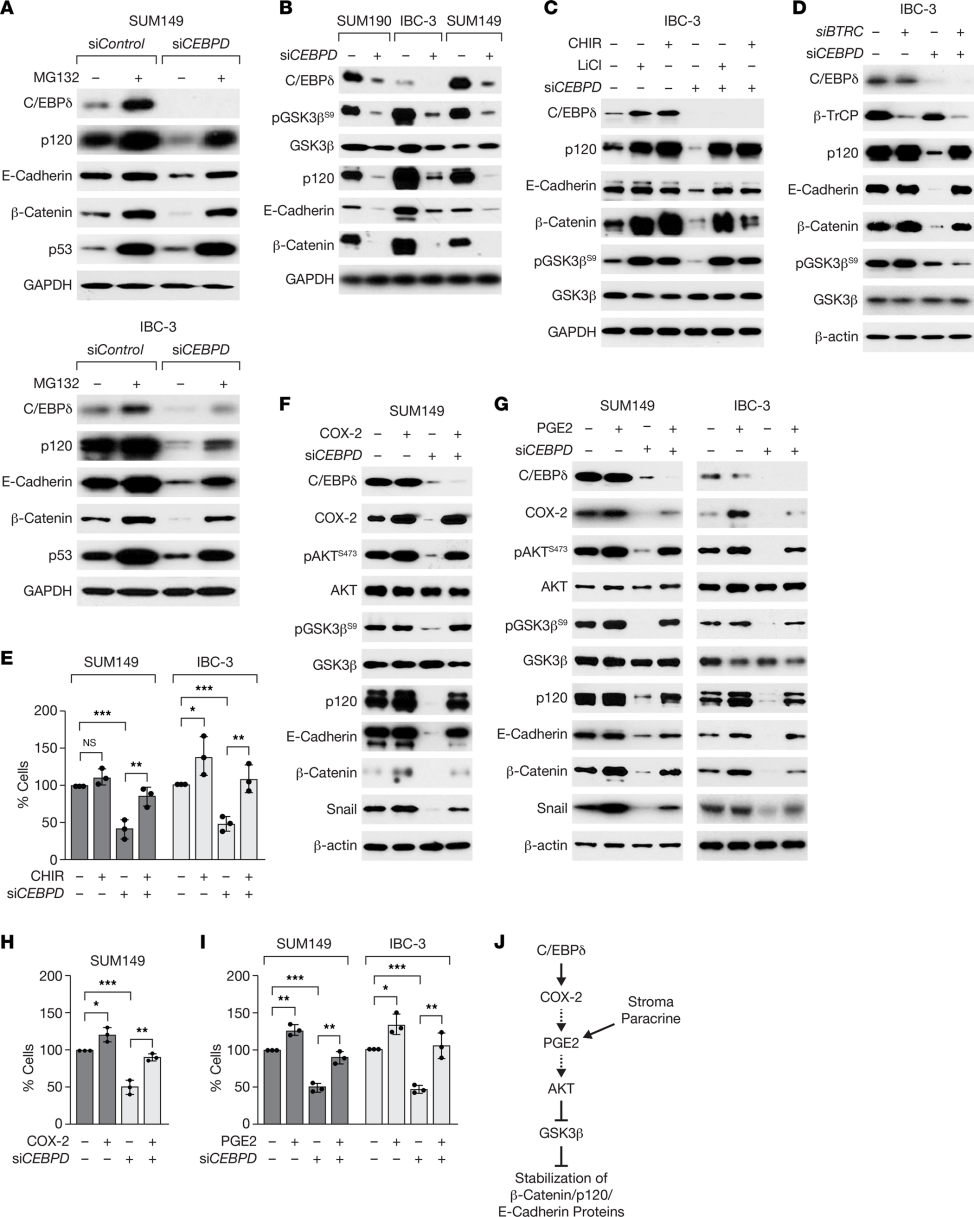
C/EBPδ promotes expression of E-cadherin complex proteins through COX-2–mediated GSK3β inhibition. (**A**) Western blot analysis of emboli from SUM149 and IBC-3 cells transfected with siRNA as indicated and treated with 20 μM MG132 for 6 hours. p53 was used as a control for MG132 treatment (83). (**B**) Western blot analysis of the indicated proteins in emboli from SUM190, IBC-3, and SUM149 cells that were transfected with control or si*CEBPD* oligos. (**C**) Western blot analysis of the indicated proteins in emboli from IBC-3 cells transfected with control (–) or si*CEBPD* oligos and treated with LiCl (10 mM) or CHIR (5 μM) for 6 hours. (**D**) Western blot analysis of the indicated proteins in emboli from IBC-3 cells transfected with control (–) or si*CEBPD* along with si*BTRC* (β-TrCP) oligos. (**E**) Analysis of the number of cells in emboli of SUM149 or IBC-3 cells that were transfected with si*Control* (–) or si*CEBPD* oligos and 24 hours later seeded in 3D for 3 days ± 1 μM CHIR (*n* = 3, mean ± SEM; *P < 0.05, **P < 0.01, ***P < 0.001). (**F**) Western blot analysis of the indicated proteins from SUM149 cells transfected with control (–) or si*CEBPD* (+) oligos and COX-2 expression plasmid followed by culture in 3D for 3 days. (**G**) Western blot analysis of the indicated proteins in SUM149 and IBC-3 emboli by cells transfected as in **A** followed by culture in 3D for 3 days ± PGE2 (1 μM). (**H**) Number of cells in SUM149 emboli as in **F** (% of control, *n* = 3, mean ± SEM; *P < 0.05, **P < 0.01, ***P < 0.001). (**I**) Number of cells in emboli of SUM149 and/or IBC-3 cells as in **G** (*n* = 3, mean ± SEM; *P < 0.05, **P < 0.01, ***P < 0.001). (**J**) Model summarizing the signaling pathway described in this study and indicating that PGE2 may be generated by autocrine or paracrine/stromal mechanisms.

Because E-cadherin/catenin mRNA levels were not altered by C/EBPδ depletion, we tested whether C/EBPδ regulated their protein stability. Treatment of emboli with the proteasome inhibitor MG132 significantly increased E-cadherin, β-catenin, and p120 protein levels in *CEBPD*-depleted cells but had comparatively less effect on these proteins in control cells (Figure 2A and Supplemental Figure 2A). E-cadherin protein stability depends in part on the formation of complexes at the cell membrane, which can be regulated by the abundance of β-catenin and p120 (32, 33), as was also demonstrated in SUM149 cells for p120 (33). The stability of β-catenin and p120 can be regulated by the serine/threonine kinase GSK3β, which targets the proteins for degradation by the Skp1-Cullin-F-box^β–TrCP^ E3 ubiquitin ligase (32, 34). In C/EBPδ-depleted IBC cell lines, the inhibitory phosphorylation on Serine 9 (Ser9) of GSK3β (35) was significantly reduced, suggesting a higher level of GSK3β activity (Figure 2B and Supplemental Figure 2B). Treatment with 2 different GSK3β inhibitors (CHIR or LiCl) rescued the protein levels of p120, β-catenin, and E-cadherin completely or partially in C/EBPδ-depleted cells (Figure 2C and Supplemental Figure 2C). In control cells, GSK3β inhibition did not affect E-cadherin levels (Figure 2C and Supplemental Figure 2C), suggesting that E-cadherin is not directly regulated by GSK3β. Similarly, silencing of the β-TrCP (*BTRC*) subunit of the E3 ligase increased the expression of E-cadherin complex proteins in C/EBPδ-depleted cells, although pGSK3β^S9^ was not rescued (Figure 2D and Supplemental Figure 2D), implicating β-TrCP in mediating their targeted degradation. Notably, GSK3β inhibition significantly rescued the ability of *CEBPD*-silenced SUM149 and IBC-3 cells to associate into emboli (Figure 2E). Taken together, these data show that C/EBPδ-mediated inhibition of GSK3β supports the accumulation of E-cadherin complex proteins and cell-to-cell adhesion.

Next, we investigated the mechanism by which C/EBPδ mediates GSK3β inhibition. GSK3β phosphorylation on Ser9 can be mediated by several kinases including AKT that can be activated by many signaling pathways such as that of prostaglandin E2 (PGE2), a downstream metabolite of the COX-2 enzyme. COX-2 is a target gene of C/EBPδ (36), correlates with AKT activation in BC (37, 38), and is highly expressed in IBC (39, 40). Indeed, *CEBPD* silencing reduced the expression of COX-2 mRNA and protein but not COX-1 protein (Supplemental Figure 2, E and F), which was rescued by C/EBPδ overexpression (Supplemental Figure 2, G and H). Along with downregulation of COX-2, *CEBPD* silencing also reduced the phosphorylation of AKT and GSK3β and the expression of Snail, which is typically induced by COX-2 signaling (41, 42) (Figure 2F). Ectopic expression of COX-2 in C/EBPδ-silenced cells rescued phosphorylation of these proteins as well as expression of E-cadherin/catenin proteins (Figure 2F). Similar results were obtained when *CEBPD*-depleted SUM149 or IBC-3 cells were treated with PGE2 (Figure 2G). Correspondingly, COX-2 overexpression (Figure 2H) or PGE2 treatment (Figure 2I) rescued the number of C/EBPδ-depleted SUM149 and IBC-3 cells associating into emboli. In summary, these data show that C/EBPδ-mediated COX-2 expression and activity led to AKT activation and GSK3β inhibition in IBC cell emboli and that this pathway contributed significantly to the expression of epithelial cadherin complex proteins and cell-to-cell adhesion in 3D (Figure 2J).

### The COX-2/GSK3β/E-cadherin pathway is conserved in a subset of BCs in vivo.

**Figure 3:**
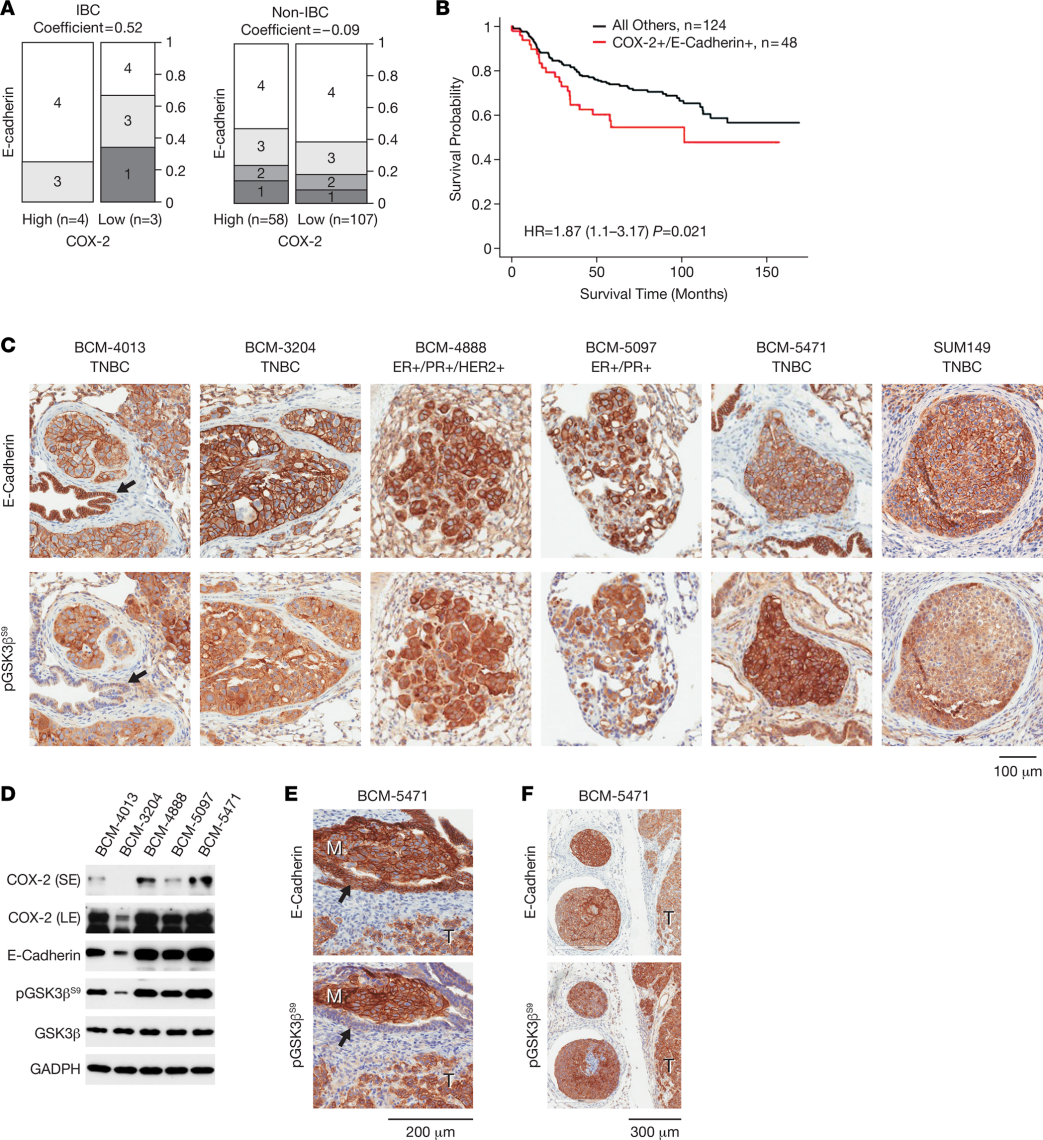
The COX-2/GSK3β/E-cadherin pathway is conserved in a subset of breast cancers in vivo. (**A**) Bar graph showing proportion of samples by different degrees of IHC staining of COX-2 and E-cadherin in IBC (*n* = 7) and non-IBC (*n* = 165) tumor tissues. Numbers 1–4 within boxes (along with dark to lighter shades of gray) denotes low to high expression levels of E-cadherin. Columns represent high (score 3–4) versus low (score 1–2) COX-2 expressing samples. Width of columns and scale denotes relative proportion of samples with different combinations of scores. “Coefficient” refers to Pearson correlation coefficient for COX-2 and E-cadherin expression. (**B**) Kaplan-Meier plot with the hazard ratio (HR) and 95% CI from a Cox regression analysis comparing patients with high expression of both, COX-2 and E-cadherin, against all other patients (reference group). Patients with high COX-2 and E-cadherin expression (denoted as COX-2^+^/E-cadherin^+^) in their tumors have a significantly decreased breast cancer-specific survival when compared with all other patients (*P* = 0.021). (**C**) Immunostaining of E-cadherin and pGSK3β^S9^ on serial sections of lung metastases from PDX primary tumors of the indicated breast cancer subtypes and an experimental metastasis by SUM149 cells. Black arrows indicate bronchial epithelium (BCM-4013). (**D**) Western blot analysis of tumor tissue extracts from the indicated PDX models. S/LE, short/long exposure. (**E**) Immunostaining as in **C** of BCM-5471 showing a micrometastasis within a mammary duct (M, metastasis; T, tumor; arrow, mouse mammary epithelium). Scale bar: 200 μm. (**F**) BCM-5471 as in **C** showing emboli-like structures next to primary tumor (T). Scale bar: 300 μm.

To assess the potential in vivo relevance of our findings, we examined COX-2 and E-cadherin expression in clinical BC specimens. We focused our analyses on E-cadherin because it is the molecule that bridges cell-to-cell contacts. Cooccurrence of high COX-2 and E-cadherin expression (scores 3–4 for both; ref. 37) was observed in 48 of 172 (28%) of the breast tumors, was overrepresented in IBC (4 of 7 or 57%) compared with non-IBC (44 of 165 or 27%) (Figure 3A), and was associated with worse BC-specific survival probability (Figure 3B). Next, we evaluated E-cadherin and pGSK3β^S9^ in 5 metastatic PDX models, 2 ER^+^/PR^+^ (BCM-4888, BCM-5097) models, of which BCM-4888 is also HER2^+^, and 3 TNBC models (BCM-4013, BCM-3204, BCM-5471), with BCM-5471 expressing the most C/EBPδ (27). By immunostaining, the primary tumors of these models (Supplemental Figure 3, A and B) — as well as spontaneous PDX lung metastases and SUM149 experimental metastases — were positive for E-cadherin and pGSK3β^S9^ (Figure 3C). Western blot analysis confirmed that all PDXs expressed these proteins and also COX-2, albeit at varying levels (Figure 3D). For further analysis, we chose BCM-5471, a basal-like subtype (43), because of the documented highest level of COX-2 (*PTGS2*) mRNA (Supplemental Figure 3C). E-cadherin and pGSK3β^S9^ staining of BCM-5471, IBC-3, and SUM149 tumors was comparable or more intense than that of ER^+^ luminal MCF-7 cells, while the basal-like, claudin-low (mesenchymal) TNBC cell lines SUM159 and MDA-MB-231 (44) were negative for E-cadherin and variable for pGSK3β^S9^ (Supplemental Figure 3D). BCM-5471 also presented with local metastases that showed strong immunoreactivity for both E-cadherin and pGSK3β^S9^, such as a metastasis within a mammary duct or parenchymal tumor cell clusters resembling large emboli next to the primary tumor (Figure 3, E and F). Bronchial epithelial cells (Figure 3C) and normal mammary epithelial cells (Figure 3E) expressed high levels of E-cadherin, as expected, but were comparatively negative for pGSK3β^S9^. However, bronchial epithelial cells in close vicinity to metastatic lung lesions often exhibited stronger pGSK3β^S9^ staining compared with more distal cells (Supplemental Figure 3E). While there can be other causes, this result is consistent with paracrine inhibition of GSK3β by factors such as PGE2. Collectively, these data indicate that coexpression of E-cadherin with pGSK3β^S9^ and COX-2 expression is observed in vivo in a subset of BCs including metastatic PDXs and could be indicative of aggressive tumor biology.

### The COX-2 inhibitor celecoxib downregulates E-cadherin protein in vitro and in vivo and reduces SUM149 tumor growth and cluster CTCs.

**Figure 4:**
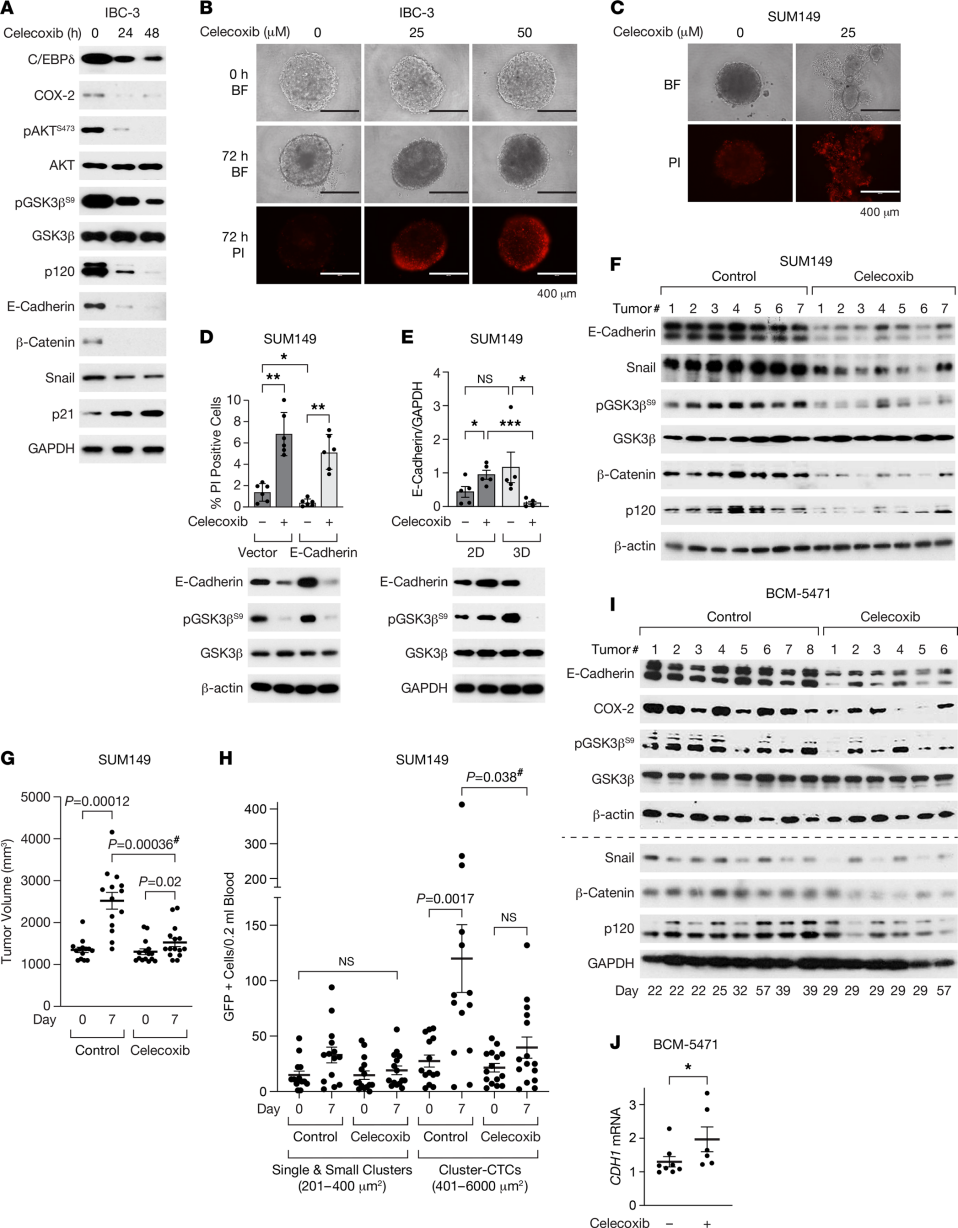
The COX-2 inhibitor celecoxib downregulates E-cadherin protein in vivo and reduces SUM149 tumor growth and cluster CTCs. (**A**) Western blot analysis of IBC-3 emboli established after 3 days of culture in 3D followed by treatment for the indicated times with 50 μM celecoxib (0 hours = 48 hours DMSO). (**B**) Images of representative IBC-3 emboli after 3 days of culture (0 hours) and the same emboli following another 72 hours with celecoxib and stained with propidium iodide (PI) to label dying cells as indicated (representative of 3 experiments; BF, bright-field). Scale bar: 400 μm. (**C**) Representative images of SUM149 cells cultured in 3D ± celecoxib for 72 hours and stained with PI (representative of 3 experiments; BF, bright-field). Scale bar: 400 μm. (**D**) Assessment of cell death by PI staining (top panel) and Western blot analysis (bottom panel) from SUM149 cells that were transfected with empty vector or E-cadherin–expressing plasmid followed by culture in 3D for 1 day and treated with celecoxib for additional 3 days (*n* = 3, mean ± SEM; *P < 0.05, **P < 0.01). (**E**) Western blot analysis (bottom panel) of SUM149 cells cultured on plastic (2D) or as emboli (3D) for 3 days followed by treatment with celecoxib for another 3 days, and quantification of E-cadherin from 5 independent experiments (*n* = 5; *P < 0.05, ***P < 0.001). (**F** and **G**) Western blot (**F**) and tumor volume (**G**) analysis of SUM149-GFP-Luc orthotopic tumors from mice fed control chow or celecoxib chow for 7 days starting at tumor volumes > 1,000 mm^3^ (*n* =14–15, paired or unpaired [indicated with #] 2-sided Wilcoxon rank-sum test). (**H**) CTC analysis of peripheral blood drawn from mice as in **F** and **G** (*n* =14–15, paired or unpaired [indicated with #] 2-sided Wilcoxon rank-sum test). (**I** and **J**) Western blot (**I**) and *CDH1* mRNA (**J**) analysis of BCM-5471 PDX tumors from mice that were fed control chow or celecoxib chow for the indicated number of days (determined by study end points) starting when tumor volumes were 300–600 mm^3^ (*n* = 6–8; *P = 0.029 by unpaired 2-sided Wilcoxon rank-sum test).

To determine the effect of pharmacological COX-2 inhibition on E-cadherin/catenin expression, we treated established in vitro emboli with celecoxib, which inhibited AKT and GSK3β phosphorylation within 24–48 hours and reduced expression of β-catenin, p120, and E-cadherin as well as Snail (Figure 4A). Celecoxib also downregulated COX-2 and C/EBPδ expression, consistent with autoregulation and positive feedback regulation, while p21^CIP1/WAF1^ expression was induced (Figure 4A). These events were followed by induction of cell death (Figure 4B and Supplemental Figure 4A). When added at the time of seeding in 3D, celecoxib prevented cell aggregation, and some cells underwent cell death by day 3 (Figure 4C and Supplemental Figure 4B). Celecoxib also downregulated ectopic E-cadherin protein (Figure 4D), which confirms that COX-2 supports E-cadherin expression at the protein level and explains why ectopic E-cadherin could not rescue cell survival in 3D (Figure 4D). However, these results contradict the previously reported upregulation of E-cadherin expression by celecoxib in SUM149 cells (39). We resolved this paradox by comparing culture conditions, which revealed that celecoxib induced E-cadherin when cells were cultured on plastic (2D) as opposed to 3D (Figure 4E). Similarly, loss of pGSK3β S9 phosphorylation was only seen in 3D. While the reasons for this difference remain to be determined, 3D culture paradigms are considered more predictive of in vivo cellular responses (45). Thus, we next evaluated the effect of celecoxib on SUM149 orthotopic primary tumor xenografts. Treatment of mice with large tumors for 7 days downregulated expression of the EMT factor Snail (Figure 4F). Nonetheless, tumors of celecoxib-treated mice exhibited significantly less pGSK3β^S9^ and E-cadherin protein (Figure 4F and Supplemental Figure 4C) as well as less p120 and β-catenin, although *CDH1* mRNA levels were higher (Supplemental Figure 4D). Despite the downregulation of E-cadherin protein, the tumor growth rate was attenuated by celecoxib (Figure 4G). Inhibition of COX-2 by celecoxib was validated by lower levels of PGE2 in the plasma and tumor tissue of treated mice (Supplemental Figure 4, E and F). Quantification of CTCs, through expression of a GFP reporter, before and after celecoxib treatment, demonstrated that cluster CTCs increased over time in untreated mice but not significantly in treated mice (Figure 4H). As an alternate non-IBC model system, we also treated BCM-5471 PDX tumors with celecoxib and again observed that E-cadherin expression was lower at the level of protein but not mRNA (Figure 4, I and J), along with reduced levels of COX-2, pGSK3β^S9^, β-catenin, p120, and Snail (Figure 4I and Supplemental Figure 4G). Taken together, these data from 3D cultures and 2 TNBC in vivo model systems show that a therapeutic effect of celecoxib was accompanied by downregulation of E-cadherin protein expression.

### Celecoxib cooperates with paclitaxel in attenuation of experimental and spontaneous lung metastases.

**Figure 5:**
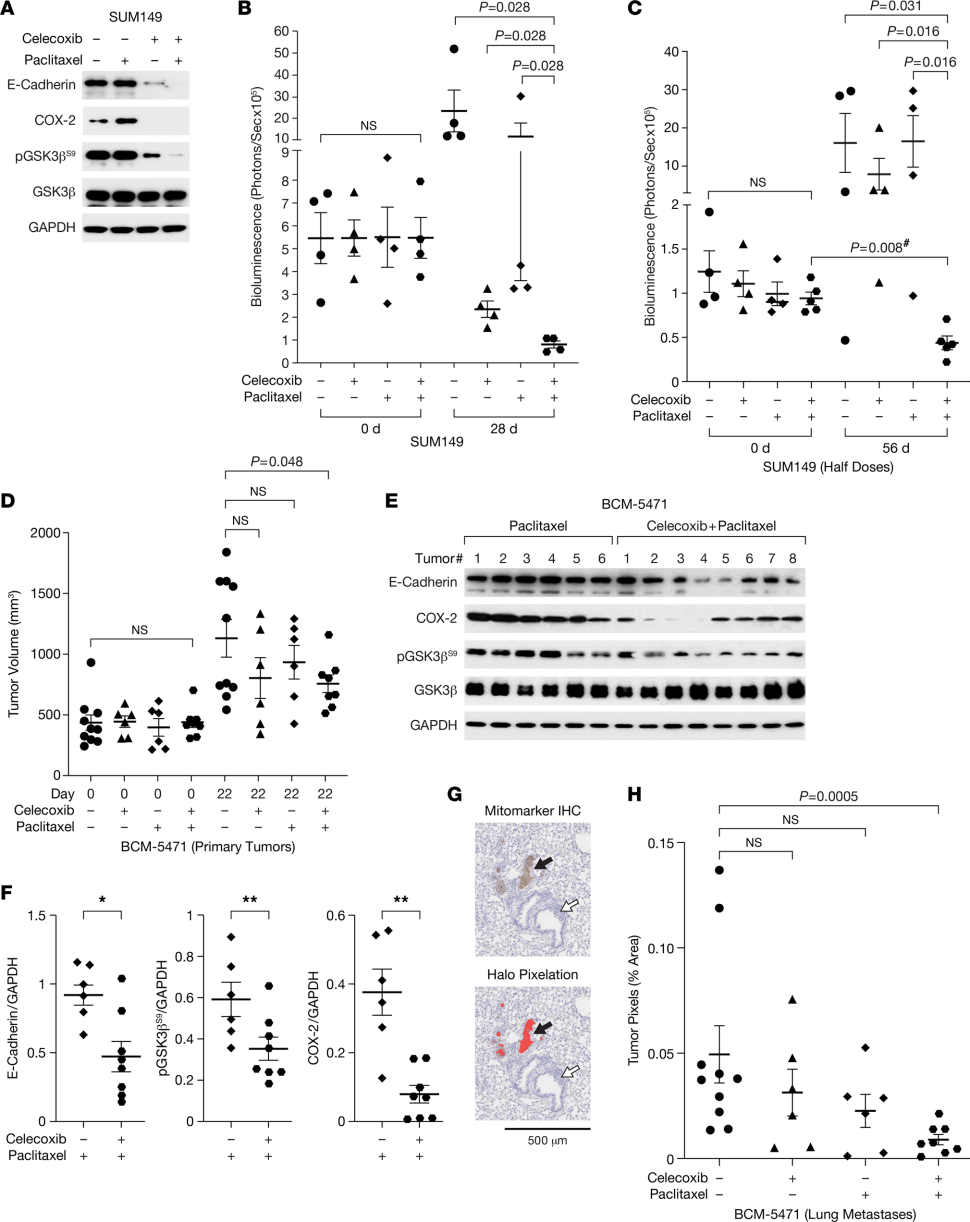
Celecoxib combination with paclitaxel attenuates experimental and spontaneous lung metastases. (**A**) Western blot analysis of the indicated proteins in SUM149 emboli after exposure to 50 μM celecoxib and/or 10 nM paclitaxel for 3 days (total time in 3D, 6 days). (**B**) Quantification of bioluminescence in the lungs of mice (*n* = 4) with experimental metastases of SUM149-GFP-Luc cells before (day 0) or after 28 days of treatment with celecoxib (1,000 mg/kg chow) and/or paclitaxel (10 mg/kg i.v.). *P = 0.028 by unpaired 2-sided Wilcoxon rank-sum test. (**C**) Quantification of bioluminescence in mice (*n* = 4–5) as in **B** after 56 days of treatment with celecoxib (500 mg/kg chow) and/or paclitaxel (5 mg/kg i.v.). *P* values as indicated by unpaired or paired (indicated with #) 2-sided Wilcoxon rank-sum test. (**D**) Tumor volume measurements of BCM-5471 PDX in mice on day 0 and 22 of treatment as in **B** (*n* = 6–10, *P* values as indicated by 2-sided *t* test). (**E**) Western blot analysis of BCM-5471 PDX tumors from mice in **D** after treatment with paclitaxel ± celecoxib. (**F**) Quantification of E-cadherin, pGSK3β^S9^, and COX-2 signals in **E** (*n* = 6–8, mean ± SEM; *P* values by unpaired 2-sided Wilcoxon rank-sum test; *P < 0.05, **P < 0.01). (**G**) Light microscope image of a mouse lung section from the experiment in **D** showing representative micrometastases immunostained with human-specific “mitomarker“ (top panel) and their pixilation by the Halo image analysis software (bottom panel). The black arrow points to a micrometastasis. The white arrow points to bronchial tissue. Scale bar: 500 μm. (**H**) Quantification of tumor cell pixels in representative sections of lungs from mice as in **G** (% of total lung area, *n* = 6–10; *P* = 0.0005 by unpaired Wilcoxon rank-sum test).

Lung metastases initiate as intravascular emboli that require E-cadherin, as has been shown through antibody-based inhibition (6). We corroborated this notion by a genetic approach in which the E-cadherin gene was deleted by inducible Cre-recombination in mouse mammary tumor cells (12) after the onset of lung colonization, which significantly reduced the tumor burden in lungs (Supplemental Figure 5A). Despite the presence of CTCs (Figure 4H), in our experience, only about 10% of mice with SUM149 xenografts developed spontaneous lung metastases. Thus, we proceeded to evaluate experimental lung metastases generated after tail vein injection of luciferase-expressing SUM149 cells. When bioluminescence imaging (BLI) confirmed lung colonies, mice were randomized to treatments. In addition to celecoxib, we also used paclitaxel after having determined the combinatorial benefit of these 2 drugs in 3D culture (Supplemental Figure 5, B and C) as well as efficient downregulation of E-cadherin and pGSK3β^S9^ (Figure 5A). Celecoxib also countered the increase in COX-2 expression seen with paclitaxel alone. At dosing as previously reported for combination treatments of TNBC models (46, 47), both paclitaxel and celecoxib monotherapy reduced BLI signal in the lungs compared with untreated mice, while the combination therapy completely eliminated bioluminescence (Figure 5B), confirmed by histological evaluation of lungs (Supplemental Figure 5D). When the doses were halved, monotherapies were no longer effective, but combination therapy significantly attenuated the BLI signal (Figure 5C). These results show that celecoxib can diminish established SUM149 experimental lung metastases and synergizes with paclitaxel treatment. The data also indicate that the 3D emboli culture paradigm modeled SUM149 cell responses in vivo. Next, we proceeded to evaluate the drug response of BCM-5471. Because this PDX model does not express a luciferase reporter, we began treatment when tumors were well established and were likely to have seeded lung metastases. Celecoxib alone and combination treatment slowed primary tumor growth to varying degrees (Supplemental Figure 5E), and the combination treatment resulted in reduced tumor volumes after 22 days of treatment (Figure 5D). Western blot analysis confirmed that tumors under combination treatment exhibited lower levels of E-cadherin, pGSK3β^S9^, and COX-2 compared with paclitaxel alone (Figure 5, E and F). Histological quantification of spontaneous micrometastases showed that the monotherapies had no significant effect but that the lungs of mice under combination treatment harbored significantly fewer tumor cells than untreated mice (Figure 5, G and H). Taken together, these data demonstrate a therapeutic benefit of celecoxib alone (SUM149) or in combination with paclitaxel (SUM149, BCM-5471) in reducing both experimental and spontaneous lung metastases by cells expressing both COX-2 and E-cadherin, and they demonstrate that this is accompanied by downregulation of E-cadherin protein levels.

## Discussion

In this study, we have mechanistically connected 2 seemingly distinct aspects in cancer biology: the role of inflammation in BC metastasis as exemplified by COX-2 signaling and the expression of E-cadherin mediating cell-to-cell adhesions. COX-2 and its downstream metabolite PGE2 are enriched in invasive BC, including IBC (39, 48), and COX-2 expression mostly correlates with ER^–^ status, advanced disease, and shorter survival probability (42). We show that COX-2 and E-cadherin are coexpressed in clinical specimens with poor survival probability and metastatic PDX models. Furthermore, the COX-2 inhibitor celecoxib downregulated E-cadherin protein while also attenuating primary tumor growth of 2 TNBC models. Celecoxib reduced cluster CTC and, especially when combined with paclitaxel, experimental and spontaneous lung metastases.

Through studies of IBC cell lines and their ability to form emboli in culture, we resolved a molecular mechanism by which COX-2 signaling supports E-cadherin protein expression via GSK3β inhibition, possibly through direct stabilization of p120 and β-catenin. This mechanism superseded regulation of *CDH1* gene expression. In response to celecoxib, expression of the EMT transcription factor Snail decreased and *CDH1* mRNA expression increased in xenograft tumors. However, E-cadherin protein was nevertheless reduced. Comparison of culture conditions showed that celecoxib interferes with E-cadherin protein stability specifically in 3D, as opposed to 2D culture on plastic. A variety of 3D culture paradigms have been established to model the tumor architecture and were shown to mimic more closely the physiological context compared with cell culture on plastic (49, 50). Even mammosphere formation as a cancer stem cell assay requires expression of E-cadherin (51). The most unique feature of “emboli culture” compared with other 3D paradigms is the mechanophysical environment (23). Our ongoing studies are addressing to what extent specific 3D culture methods affect signaling pathways. However, in the current report, we demonstrate that the “emboli culture” method replicates the effect of celecoxib seen in vivo.

We also identified C/EBPδ as a tumor cell intrinsic factor that can initiate the COX-2/E-cadherin pathway. C/EBPδ is most highly expressed during the first, inflammatory phase of postpartum mammary gland involution (52) and again in the fully involuted stage (53). These conditions, which also involve COX-2 signaling, promote the risk of aggressive postpartum BC including IBC (54–56). However, COX-2 can also be induced by C/EBPδ-independent pathways (57). Moreover, the tumor microenvironment can be an alternate source for PGE2 (54). Indeed, elevated stromal expression of COX-2 was seen in canine IBC compared with non-IBC (40, 58). Thus, in addition to tumor cell intrinsic C/EBPδ and COX-2, it is conceivable that the tumor microenvironment could also be a source of PGE2 or other signaling pathways that may promote E-cadherin adhesions through inhibition of GSK3β.

A positive correlation of COX-2 and E-cadherin proteins is supported by clinical data from a large collection of BC specimens (59) and from a cohort of canine IBC (60). The latter study also reports that this correlation was specific for protein and not mRNA, corroborating our observations and highlighting the importance of understanding mechanisms operating at the level of protein. Many studies on EMT and its transcription factors have focused on target gene mRNA expression and may have missed additional layers of regulation at the level of the protein (61). A recent guide on the definition of EMT and EMP notes the paucity of investigations that address protein expression and use relevant models such as 3D culture (22). While complete EMT is among the mechanisms that may explain increased metastasis in some cancer types and model systems, EMP has been recognized as an important alternative pathway (62), and disruption of cell-to-cell adhesion is a new frontier for targeting metastatic cancer cells (63, 64). Indeed, an important element of plasticity may be the leveraging of multiple layers of regulation from gene transcription to protein stability. While *CDH1* (E-Cadherin) mRNA expression can be downregulated by EMT transcription factors, residual levels of transcript may be sufficient for maintenance of cell-to-cell adhesion by mechanisms that increased protein stability. This concept may reconcile our data with previous reports that COX-2 expression in MCF-7 cells promotes EMT (65, 66). In these studies, E-cadherin expression was not completely lost and may be the result of a balance of pathways operating at the mRNA and protein levels to maintain plasticity. In addition, the pathway described in our study was derived from analyses of HR^–^ models (2 TNBC, 1 HER2^+^). Thus, it is possible that the context of intrinsic BC subtypes, which represent “unique diseases” (67), modulates the effect of COX-2 on E-cadherin expression, in addition to intersection with other signaling pathways. For example, it has been shown that COX-2/PGE2 inhibit EMT at the juncture of hepatocyte growth factor (HGF) and TGF-β signaling (68, 69). We obtained comparable results with 2 different IBC cell lines in vitro (TNBC SUM149, HER2^+^ IBC-3), and a TNBC cell line (SUM149) and PDX model (BCM-5471) in vivo. Future studies will have to determine to what extent the BC subtypes within ductal carcinomas may modulate the relationship of COX-2 signaling, E-cadherin protein expression, and metastasis.

Both antibody-based inhibition (6, 70) and genetic deletion reveal a critical role for E-cadherin in promoting cancer cell survival and metastasis in invasive ductal BC (12, 16). Furthermore, knockdown of E-cadherin impaired primary tumor growth of SUM149 and Mary-X IBC xenograft models, and 4T1 mouse TNBC, as well as experimental metastases of SUM149 (11). Conversely, ectopic expression of E-cadherin promotes metastasis of mesenchymal and luminal-epithelial cell lines (16, 71). These reports highlight the clinical potential of targeting E-cadherin expression and corroborate our observation that downregulation of E-cadherin by celecoxib was accompanied by attenuated tumor growth, reduction in cluster-CTCs and lung metastases, and increased sensitivity to paclitaxel treatment.

COX-2/PGE2 is an attractive therapeutic target, as it acts on multiple cell types within cancers and can also be induced by conventional chemotherapy (54, 72). Combination of celecoxib with other chemotherapies including taxanes have been tested in preclinical models and clinical trials and showed some indication of efficacy (73, 74). However, these trials revealed an unmet need for biomarkers to better identify which cancers may respond to combination treatments that include celecoxib (75, 76). While downregulation of E-cadherin may not always be necessary for response to celecoxib, our study shows that COX-2 can maintain cell-to-cell adhesions in HR^–^ aggressive BC cells through GSK3β inhibition. The net effect of COX-2 on cancer biology will depend on substrate availability, the activity of enzymes downstream of COX-2, and expression of transporters and receptor combinations, all of which pertains to cancer cells as well as various cell types of the tumor microenvironment (54, 77). Thus, it will be important to identify molecular markers that report the relevant prostaglandin receptor activation along with E-cadherin expression. Phosphorylation status of GSK3β may be an interesting candidate. Future clinical studies will have to address if combined evaluation of E-cadherin protein, COX-2, and pGSK3β^S9^ could contribute to the identification of patients with metastatic BC who may benefit from combination therapies that target the PGE2 signaling pathway.

## Methods

### Antibodies.

Antibodies were obtained from the following sources, unless indicated otherwise: Cell Signaling Technology (pSTAT3^Y705^, 9145; STAT3, 4904; Cleaved Notch-1 [NICD], 2421S; E-cadherin, 3195 [24E10], 5296 [32A8]; α-catenin, 3236S; β-catenin, 9562S; COX-1, 4841S; COX-2, 12282S; pGSK3β^S9^, 9323T; GSK3β, 9315S; pAKT^S473^, 9271S; AKT, 4691; p21, 2947S; Snail, 3879S; pSMAD2, 3108; pSMAD3, 9520; SMAD2, 5339; SMAD3, 9523T; SMAD4, 38454; β-TrCP, 4394; p53, 2524S; and Cleaved Caspase-3, 9664), eBiosciences (CD44-PE, 12-0441-82, clone IM7; CD24-FITC, 11-0247-41, clone eBioSN3; and CD24-APC, 17-0247-42, clone eBioSN3), Abcam (β-actin, ab6276; CEBPβ, ab32358), Santa Cruz Biotechnology Inc.(CXCR4, sc-9046; GAPDH, sc-47724; and CEBPδ, sc-135733), Rockland (α-tubulin, 600-401-880), and BD Biosciences (p120, 610133).

### Cells, culture, and reagents.

MCF-7, T47D, MDA-MB-468, MDA-MB-231, and SKBR3 cells were obtained from ATCC; SUM149 and SUM159 cells originated from Asterand Bioscience. IBC-3 and KPL-4 cells were provided by Wendy A. Woodward (MD Anderson Cancer Center [MDACC], Houston, Texas, USA) and Junichi Kurebayashi (Kawasaki Medical School, Kurashiki, Japan), respectively. Brain tropic SUM190-BR cells were provided by Patricia Steeg (NCI, Bethesda, Maryland, USA), and SUM149 derivatives were provided by Jangsoon Lee (MDACC) and Stanley Lipkowitz (NCI). Cell lines were authenticated approximately every 2 years and last in 2022 by GenePrint 10 (Promega) and tested for *Mycoplasma* infection annually by quantitative PCR (qPCR). Cells were cultured in a 5% CO_2_ incubator at 37°C in media with 100 units/mL penicillin and 100 μg/mL streptomycin as follows: MCF-7, MDA-MB-231, and MDA-MB-468 in DMEM, MCF-7 also with 1 mM sodium pyruvate; T47D in ATCC-formulated RPMI-1640 Medium (catalog 30-2001) with 0.2 units/mL bovine insulin (Sigma-Aldrich, I0516); SUM159 in RPMI with 2 mM glutamine, 10 mM HEPES, 1 mM sodium pyruvate, 1***×*** nonessential amino acids (Thermo Fisher Scientific, 11140-050), and 55 μM β-mercaptoethanol (Thermo Fisher Scientific, 21985-023); SKBR3 cells in McCoy’s 5A Medium Modified (Thermo Fisher Scientific, 16600-082); SUM149, IBC-3, and SUM190-BR in Ham’s F-12 media (Thermo Fisher Scientific, 31765092) with 1 μg/mL hydrocortisone and 5 μg/mL Insulin; and KPL-4 cells in DMEM/F12/GlutaMax. Fetal bovine serum (FBS) was added at 10% except for SUM159 (5%). Cell culture grade chemicals were from Sigma-Aldrich unless indicated otherwise.

Celecoxib (NDC-59762-1517-1) and paclitaxel (NDC-0703-3213-01) were purchased from the NIH Pharmacy; carboplatin (catalog S1215), prostaglandin E2 (catalog S3003), and CHIR-99021 (catalog S2924) were from Selleck Chemicals; Doxorubicin (catalog D1515), MG132 (catalog 474790), and propidium iodide (PI; catalog P4170) were from MilliporeSigma. DMSO (MilliporeSigma, D-2650) was used as vehicle control in all experiments.

### PGE2 measurement by ELISA.

Prostaglandin E2 in blood plasma and tumor tissues was quantified using a commercially available kit (Cayman Chemical, 514010). As previously described (46), fresh tumor tissue was homogenized and lysed in buffer containing 50 mM Tris-HCl (pH 7.5), 150 mM NaCl, 1% Triton-X100 supplemented with 10 μL/mL protease inhibitor cocktail. Values were normalized to total protein concentration (tumors) or volume of plasma.

### 3D culture assay.

In vitro emboli formation was carried out as described (23). Briefly, cells were trypsinized 24 hours after nucleofection (if applicable); 100,000 cells were seeded in 6-well ultra-low attachment plates (Corning, 3471) in medium containing 2.25% PEG8000 and gently rocked at approximately 40 rpm for 3–4 days or as indicated. To isolate emboli, cultures were centrifuged at 27*g* for 1 minute with PBS, treated with TrypLExpress (Thermo Fisher Scientific, 12604-013) for 5–10 minutes and neutralized with cell culture medium. Cells were counted with a Countess (Thermo Fisher Scientific) using trypan blue dye exclusion. Unless indicated otherwise, all analyses of emboli were conducted after 4 days in 3D culture. For assessment of cell death within established emboli, these were generated first by seeding 10,000 cells per well in 96-well plates (Nexcelom Biosciences, ULA-96U-010), cultured and treated as indicated, followed by addition of PI (0.5 μg/mL) for 30 minutes and imaging with an EVOS FL microscope. For quantification, emboli were harvested as above; cells were transferred at 10,000 cells/well in 96-well plates. Six hours later, they were treated with PI for 30 minutes and analyzed by Direct Cell Counting (Celigo, Nexcelom).

### Invasion assay.

Cellular invasion through Matrigel was carried out using Corning BioCoat-growth factor–reduced 24-well plates according to the manufacturer’s protocol (Corning, 354483). Briefly, SUM149, IBC-3, and KPL-4 cells were nucleofected with control or *CEBPD* siRNA oligos. Seventy-two hours later, 5 × 10^4^ cells in serum-free medium were placed in the chamber and immersed in 24-well plates with serum-containing medium and incubated at 37°C for 8 hours. After fixing with 4% formaldehyde for 2–5 minutes, followed by methanol for 10–15 minutes, the cells were stained with crystal violet for 15 minutes. Migrated cells on the entire surface of the membrane were viewed under the microscope and counted manually by an investigator who was blinded to the experiment.

### Flow cytometric analysis.

About 2 ***×*** 10^5^ cells per sample were blocked using Purified NA/LE Rat anti–mouse CD16/CD32 (clone 2.4G2) antibody (BD Biosciences, 553140) followed by incubation with 1 μL of specific antibodies for 30 minutes on ice in the dark. Isotype specific antibodies and/or OneComp eBeads (eBiosciences, 01-1111-42) were used as negative controls. Cells were washed twice with ice-cold PBS containing 0.02% sodium azide, resuspended in DPBS/0.1% BSA, and analyzed with a BD FACSCanto II Analyzer and FlowJo software (FlowJo). At least 30,000 viable events per sample were collected for analysis.

### Generation of cells with stable or Dox-inducible shRNA expression.

For stable shRNA expression, SUM149 cells were infected with pDEST lentiviral vector expressing sh*CEBPD* or *GFP*-targeting (GCAAGCTGACCCTGAAGTTCAT) sh*Control* RNA, packaged with MISSION Packaging Mix (MilliporeSigma, SHP001), and selected by G418. SUM149 and IBC-3 cells with Dox-inducible shRNA expression were first infected with CMV-Luciferase-2A-GFP (Neo) (GenTarget Inc., LVP403) virus and selected as per instructions. Subsequently, cells were infected with SMARTchoice lentivirus from Dharmacon (Nontargeting control, VSC11656; CEBPD shRNA, V3SH11252-226035621) and selected per instructions. For shRNA induction, cells were treated with Dox as indicated. The sequences of the *CEBPD* siRNA and/or shRNA used in this study can be found in Supplemental Figure 6.

### Transient expression and silencing of gene expression.

pcDNA3.1-hPTGS2-2flag (78) was a gift from Jun Yu (Addgene plasmid 102498; http://n2t.net/addgene:102498; RRID:Addgene_102498). siRNA-mediated silencing was by nucleofection with AMAXA technology essentially as described (79). All experiments included nonspecific siRNA (–) as control. Scrambled siRNA was used for most experiments (sense 5′-CGUACGCGGAAUACUUCGAUUdTdT-3′), *GFP* oligonucleotides were used alternatively (sense 5′-CAAGCTGACCCTGAAGTTC-3′). Unless indicated otherwise, *CEBPD* siRNA#1 (sense: 5′-UCGCCGACCUCUUCAACAGTT-3′), *CEBPD* siRNA#2 (sense: 5′ -CCACUAAACUGCGAGAGA-3′), *CEBPB* siRNA#1 (sense: 5′-GUGGUGUUAUUUAAAGAAGAAACGT-3′), and *CEBPB* siRNA#2 (sense: 5′-AGAUGAAUGAUAAACUCUCUGCUTC- 3′) were used at 1:1 ratio. β-TrCP was silenced by *BTRC* siRNA (sense: 5′-GUGGAAUUUGUGGAACAU-3′). For each experiment, the efficiency of silencing was assessed by Western blotting and/or qPCR analysis.

### Western blot analysis.

Whole cell extracts were prepared by lysing the cells or emboli with RIPA buffer (25 mM Tris [pH 8.0]; 50 mM NaCl; 1 mM EDTA; 0.5% NP40; 0.5% sodium deoxycholate; 0.1% SDS; 10 μL/mL protease inhibitor cocktail, MilliporeSigma, P8340; 10 μL/mL phosphatase inhibitor cocktail #2, MilliporeSigma, P5726; 10 μL/mL phosphatase inhibitor cocktail #3, MilliporeSigma, P0044). Tumor extracts were prepared as described (27). Protein concentrations were measured by BCA assay (Thermo Fisher Scientific, 23225). About 10–20 μg protein was loaded onto NOVEX WedgeWell 4%–20% Tris-glycine gels, and Western blot analyses were carried out as described (79). Representative data are shown and were repeated at least 3 times each.

### RNA isolation and qPCR.

Total RNA from cell lines and tumor tissues was purified by GeneJET RNA purification kit (Thermo Fisher Scientific, K0732), and cDNA was synthesized using Superscript Reverse Transcriptase III (RT) according to manufacturer’s instructions (Invitrogen, 18080044). PCR was carried out with Fast SYBR Green master mix (Applied Biosystems, 4385612) using the 7500 Fast Real-Time PCR instrument (Applied Biosystems); the relative expression levels were measured using the relative quantitation ΔΔCt method and normalized to *RPLP0*. Data are from 3 independent biological replicates, each assayed as triplicates. For the primer details, see Supplemental Table 1.

### PDX and mice.

Tissue sections of primary PDX tumors (transplant generation 6–13) and lungs with metastases were obtained from the NCI-CCR Breast Cancer PDX Biobank. All PDX models were previously established and characterized at Baylor College of Medicine. Comprehensive description of pathology and genetic characterization of these models is available at https://pdxportal.research.bcm.edu/pdxportal (43). BCM-5471 was propagated in NOD/SCID/ILIIrg^−/−^ (NSG) mice (NCI) essentially as described (43). Experiments were performed with transplant generations 9–10. Tumor volumes were calculated as V = (W^2^ × L)/2. Celecoxib was provided in powder feed (AIN-93G, Envigo) at 1,000 mg/kg chow as described (46). Paclitaxel was administered i.v. at 10 mg/kg, once a week. Ground chow without celecoxib and injection of vehicle (50:50 ethanol/Kolliphor to 5 parts saline) were used as controls. Treatments were started when tumors were established (300–800 mm^3^) and for the indicated durations.

### SUM149 xenografts and CTC analysis.

SUM149-GFP-Luc cells (3 ***×*** 10^6^) were injected into the inguinal fat pads of 9- to 17-week-old female NSG mice. When tumors reached about 1,100–2,000 mm^3^ volume, treatment started with celecoxib (1,000 mg/kg of chow) or normal powder feed for 6–7 days. Mice were randomized to treatment based on tumor size. About 200 μL blood was collected from the tail vein before and after the treatment. Erythrocytes were lysed using ACK lysing buffer (Lonza, BP10-548E), and remaining cells were washed with PBS and suspended in 200 μL PBS, seeded into 2 wells (Corning, 655090), and analyzed for GFP^+^ cells using Celigo Imaging Cytometer (Nexcelom Bioscience). To clearly identify cluster CTCs, we gated for 3 different populations based on the determination that single SUM149 cells were approximately 200 μm^2^ in area: < 201 μm^2^ area to denote debris and some single CTCs; 201–400 μm^2^ to label single CTCs and small aggregates of GFP^+^ cells; 401–6,000 μm^2^ area to denote definitive CTCs clusters.

### Experimental metastasis assays.

SUM149-GFP-Luc cells (2 ***×*** 10^6^ to 3 ***×*** 10^6^ in PBS) were injected i.v. into 6- to 8-week-old female nu/nu mice (The Jackson Laboratory and NCI). Mice were monitored biweekly for bioluminescence and randomized by bioluminescence intensity for treatment with celecoxib (1,000 mg/kg chow) and paclitaxel (10 mg/kg) 5 times for 10 days plus 2 weekly doses (Figure 5B) or with celecoxib (500 mg/kg chow) and paclitaxel (5 mg/kg) once per week for 8 weeks (Figure 5C). In vivo BLI (IVIS Spectrum imager, PerkinElmer Inc.) was performed essentially as described (46). Bioluminescence signals were quantified by Living Image (version 4.3.1, PerkinElmer Inc.), implementing standard regions of interest (ROI) drawn over the metastatic region. MMTV-PyMT mouse mammary tumor cells with homozygous floxed *Cdh1* alleles encoding E-cadherin (E-cad^fl/fl^) and a mT/mG-Cre reporter transgene were transfected with adenoviruses expressing a tamoxifen-activatable Cre recombinase–estrogen receptor domain fusion protein (CreER) or vector control (WT) as described (12). Cells were injected as small clusters (about 2 ***×*** 10^5^ cells) into 6- to 8-week-old NSG mice (12). One week later, all mice were injected with tamoxifen (100 μL of a 2 mg/mL stock) to delete E-cadherin and induced mGFP expression in cells with E-cad^fl/fl^; CreER cells. Three weeks later, lungs were harvested, and the metastases as red and/or green fluorescent foci were counted, in a blinded manner, under a dissection microscope. These experiments were performed in accordance with protocols approved by the Johns Hopkins Medical IACUC.

### Histological analysis of tumor and lung tissue.

IHC was performed with primary antibodies for E-cadherin at 1:400 (Cell Signaling Technology, 3195), pGSK3β^S9^ at 1:100 (Abcam, ab75814), and C/EBPδ at 1:100 (Santa Cruz Biotechnology Inc., sc-135733) with isotype control rabbit monoclonal IgG (Cell Signaling Technology, 3900); and NUMA1, which is specific for human cells (80), was used at 1:00 (Lifespan, LS-B11047) with polyclonal IgG (Abcam, ab37415) as negative control. For quantification of SUM149 lung metastases, four 5 μm sections, 100 μm apart, were stained with H&E and evaluated by a veterinarian pathologist blinded to the experiment. For the quantification of PDX lung metastases, sections were stained with a human-specific anti-mitochondria antibody (“mitomarker”; Abcam, ab79479), and scanned slides were analyzed with Halo-imaging software to quantify tumor cell area per total lung area of the most representative section.

### Patient survival analysis.

IHC of COX-2 and pAKT protein expression in 248 human breast tumors (collectively representing 17 IBC, 58 TNBC, 42 HER2^+^, 145 ER^+^, and 102 ER^–^ tumors) was previously reported (37). This research has previously been approved by the NIH Office of Human Subjects Research Protections (OHSRP, 2248) and followed the ethical guidelines set by the Declaration of Helsinki. IBC samples, 6 TNBC and 1 HER2^+^, were classified as described (81). E-cadherin IHC (Dako [M3612] antibody at 1:100) was available for 172 of these tumors, collectively representing 7 IBC, 42 TNBC, 31 HER2^+^, 98 ER^+^, and 73 ER^–^ tumors. Protein expression in the tumor epithelium was scored as negative, low, moderate, or high and then categorized into low (negative to low) and high (moderate to high) for correlation and survival analysis, as previously described (37). We performed a Pearson correlation test to evaluate relationships between protein marker expression and tumor characteristics. We used Cox proportional hazards regression to estimate hazard ratios (HRs) with 95% CIs to assess the association between marker expression and BC survival. Survival curves were generated using Kaplan-Meier plots.

### C/EBPδ immunostaining in IBC patient tissues.

IBC tissues were drawn from the IBC registry at MDACC as described (82). One section per specimen was stained from mastectomies of 39 patients clinically characterized as IBC who had not achieved complete pathological response after primary systemic treatment. The specimen represented the following subtypes: 25 ER^+^/HER2^–^, 3 ER^+^/HER2^+^, 2 ER^–^/HER^+^, 9 TNBC, and 1 undefined. Of the analyzed sections, 14 specimens presented with emboli in the tumor parenchyma. The data analysis for this research was approved by the IRB of the MD Anderson Cancer Center. IHC of C/EBPδ was performed as described with monoclonal antibody 92.69 (28).

### Statistics.

Unless stated otherwise, quantitative data were analyzed by the 2-tailed unequal variance *t* test and are shown as the mean ± SEM. The number of samples (*n*) refers to biological replicates. *P* values less than 0.05 were considered significant. Exact *P* values are provided for measurements of tumor volumes and quantification of tumor cells in vivo.

### Study approvals.

Research on patient material has previously been approved by the NIH OHSRP (no. 2248) and followed the ethical guidelines set by the Declaration of Helsinki. For studies with animals, NCI-Frederick is accredited by Association for Assessment and Accreditation of Laboratory Animal Care International (AALACi) and follows the Public Health Service Policy for the Care and Use of Laboratory Animals. Animal care was provided in accordance with the procedures outlined in the *Guide for the Care and Use of Laboratory Animals* (National Academies Press, 2011) including those pertaining to studies of neoplasia (National Research Council, 1996). All experiments were conducted under protocols approved by the IACUC at NCI-Frederick.

## Author contributions

KB and ES designed research. KB, DKP, SS, SWS, LM, VP, and KC conducted experiments and acquired data.KB, SS, SWS, SK, WT, VP, SA, AJE, DKP, and ES analyzed data. SK, AJE, NTU, and SA provided reagents. KB, DKP, and ES wrote the manuscript. KB, SK, AJE, NTU, SA, DKP, SWS, and ES edited the manuscript.

## Supplementary Material

Supplemental data

## Version 1 02/09/2023

In-Press Preview

## Version 2 03/22/2023

Electronic publication
