# Supplementary material for: Stabilization of E-cadherin adhesions by COX-2/GSK3β signaling is a targetable pathway in metastatic breast cancer
Source: JCI Insight. 2023 Mar 22;8(6):e156057. doi: 10.1172/jci.insight.156057 (PMC10070121; doi:10.1172/jci.insight.156057)
Supplement: Supplemental data [file jciinsight-8-156057-s208.pdf]

SUPPLEMENTAL INFORMATION

Stabilization of E-cadherin adhesions by COX-2/GSK3β signaling is a targetable pathway in metastatic breast cancer

Kuppusamy Balamurugan[1]*, Dipak K. Poria[1], Saadiya W. Sehareen[1], Savitri Krishnamurthy[2], Wei Tang[3], Lois McKennett[4], Veena Padmanaban[5,6], Kelli Czarra[4], Andrew J. Ewald[5], Naoto T. Ueno[2], Stefan Ambs[3], Shikha Sharan[1], and Esta Sterneck[1]*

[1] Laboratory of Cell and Developmental Signaling, Center for Cancer Research (CCR), National Cancer Institute (NCI), Frederick, MD, USA

[2] Morgan Welch Inflammatory Breast Cancer Research Program and Clinic, The University of Texas MD Anderson Cancer Center, Houston, TX, USA

[3] Laboratory of Human Carcinogenesis, CCR, NCI, Bethesda, MD, USA

[4] Laboratory Animal Sciences Program, Leidos Biomedical Research Inc., Frederick National Laboratory for Cancer Research, Frederick, MD, USA

[5] Departments of Cell Biology and Oncology, Sidney Kimmel Comprehensive Cancer Center, Johns Hopkins University School of Medicine, Baltimore, MD, USA

[6] Present Address: Laboratory of Systems Cancer Biology, The Rockefeller University, New York, NY, USA

*Corresponding authors:

Esta Sterneck, sternecg@mail.nih.gov, tel.301-846-1471

Kuppusamy Balamurugan, kuppusamyb@mail.nih.gov, tel.301-846-5257

National Cancer Institute, 1050 Boyles Street, Frederick, MD 21702-120, USA

CONTENT:

- **Supplemental Tables S1**
- **Supplemental Figures S1-S6**

Supplemental Table S1. Primers used for qPCR analysis

GENE	FORWARD	REVERSE
CDH1	5’-AGCAGAACTAACACACGGGG-3’	5’-ATACCGGGGGACACTCATGA-3’
CDKN1A	5’-ACCATGTGGACCTGTCACTGT-3’	5’-TTAGGGCTTCCTCTTGGAGAA-3
CEBPD	5’-GCCATGTACGACGACGAGA-3’	5’-TTGCTGTTGAAGAGGTCGG-3’
CTNNA1	5’-GGCAGCCAAAAGACAACAGG-3’	5’-TTACGTCCAGCATTGCCCAT-3’
CTNNB1	5’-TTGAAGGTTGTACCGGAGCC-3’	5’-GCCACCCATCTCATGTTCCA-3’
CTNND1	5'-TTGAGTGGGAATCGGTGCTC-3'	5'-AGGAGGTCAGCTATGGCAGA-3'
GAPDH	5’-AAGGTCGGAGTCAACGGATTTG-3’	5’-CCATGGGTGGAATCATATTGGAA-3’
IL6	5’-ACAAATTCGGTACATCCTC-3’	5’-GCAGAATGAGATGAGTTGT-3’
PTGS2	5’-GCTGTGGGGCAGGAAGTC-3’	5’-TTGGAATAGTTGCTCATCACC-3’
RPLP0	5’-GCAATGTTGCCAGTGTCTGTC-3’	5’-GCCTTGACCTTTTCAGCAAGT-3’

Supplemental Figure S1

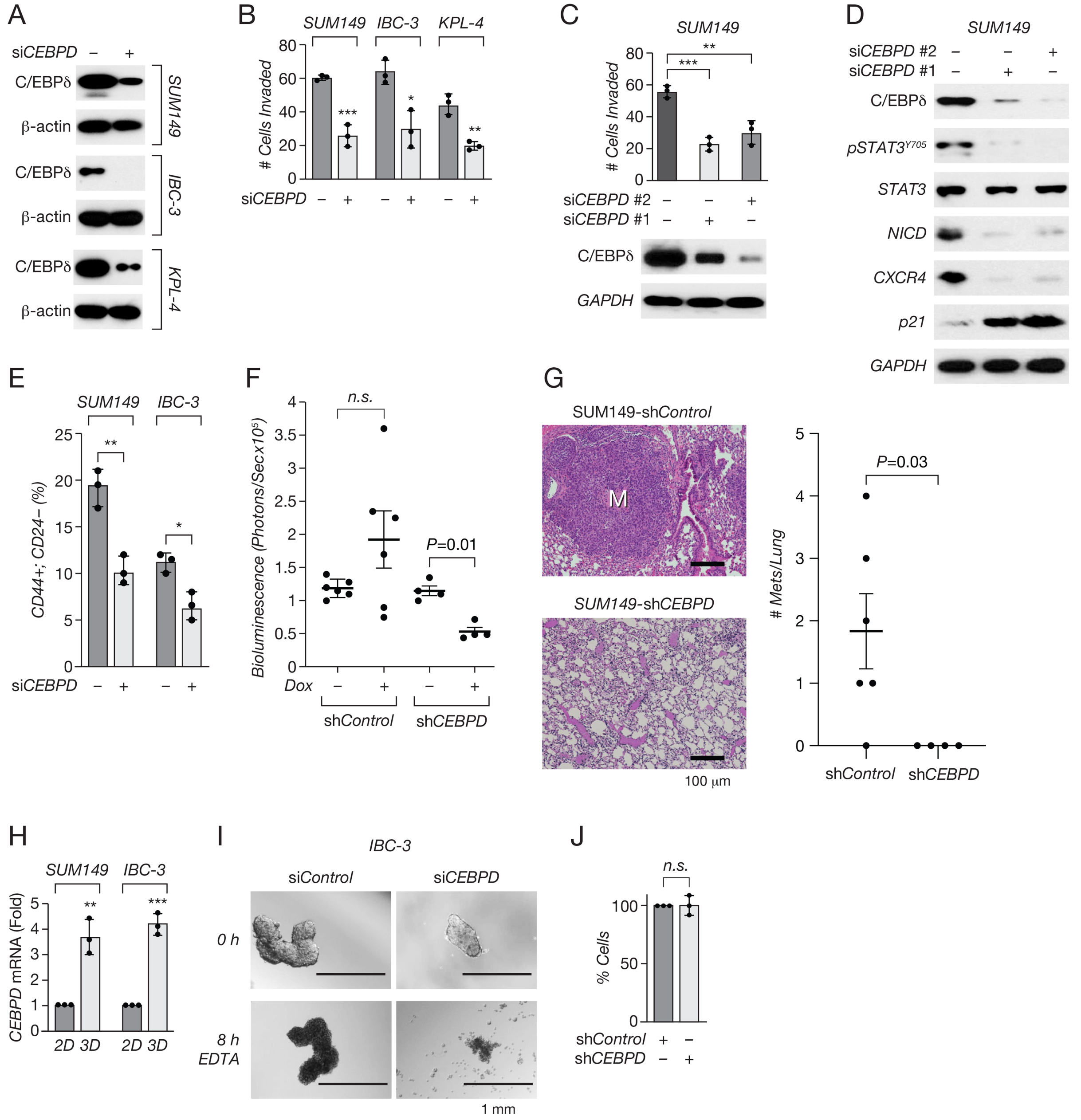

Supplemental Figure S1. C/EBPδ promotes malignant phenotypes in IBC cells. (**A**) Western analysis of C/EBPδ expression in SUM149, IBC-3 and KPL-4 cells 72 h after transfection with si*Control* (-) or si*CEBPD* oligos (+). (**B**) Quantification of the number of cells invaded through transwell Matrigel by SUM149, IBC-3 and KPL-4 cells transfected with si*Control* (-) or si*CEBPD* (+) oligos (n=3, mean±SEM; **P*<0.05, ***P*<0.01, ****P*<0.001 compared to si*Control*). (**C**) Quantification of the number of cells invaded through transwell Matrigel by SUM149 cells transfected with si*Control* (-) or two independent si*CEBPD* (+) oligos (n=3, mean ±SEM, ***P*<0.01****P*<0.001 compared to si*Control*). Western analysis of C/EBPδ expression is shown below. (**D**) Western analysis of the indicated proteins from SUM149 cells transfected with control (-) or two independent si*CEBPD* (+) oligos. (**E**) Flow cytometric quantification of cells with CD44+:CD24– cell surface markers among SUM149 and IBC-3 cells transfected with control (-) or si*CEBPD* (+) oligos followed by culture in 2D for 3 days (n=3; mean ±SEM, **P* <0.05, ***P*<0.01). (**F**) Quantification of bioluminescence (total Flux) in the lungs of mice with experimental metastases from SUM149 cells with doxycycline (Dox)-inducible shRNAs before (-) and 4 weeks after (+) treatment with Dox (n=4 and 6, ***P*<0.01 by two-sided paired t-test; n.s., not significant). (**G**) Light microscope images (left) of Hematoxylin and Eosin stained sections of lungs from mice as in panel F (M, metastasis) and quantification (right) of the number of tumor cell colonies, Mets (n=4 and 6, P=0.03 by unpaired two-sided Wilcoxon test). (**H**) qPCR analysis of *CEBPD* mRNA in SUM149 and IBC-3 cell lines that were cultured on plastic (2D) or as emboli (3D) for 4 days as in Figure 1C (n=3, mean ±SEM, ***P*<0.01, ****P*<0.001 compared to 2D). (**I**) Images of representative emboli from IBC-3 cells that had been transfected with control or si*CEBPD* oligos taken before and after treatment of the same embolus with EDTA for 8 h. (**J**) Quantification of the number of cells in 3 day-old emboli as in Figure 1I before treatment (n=3; n.s., not significant).

Supplemental Figure S2

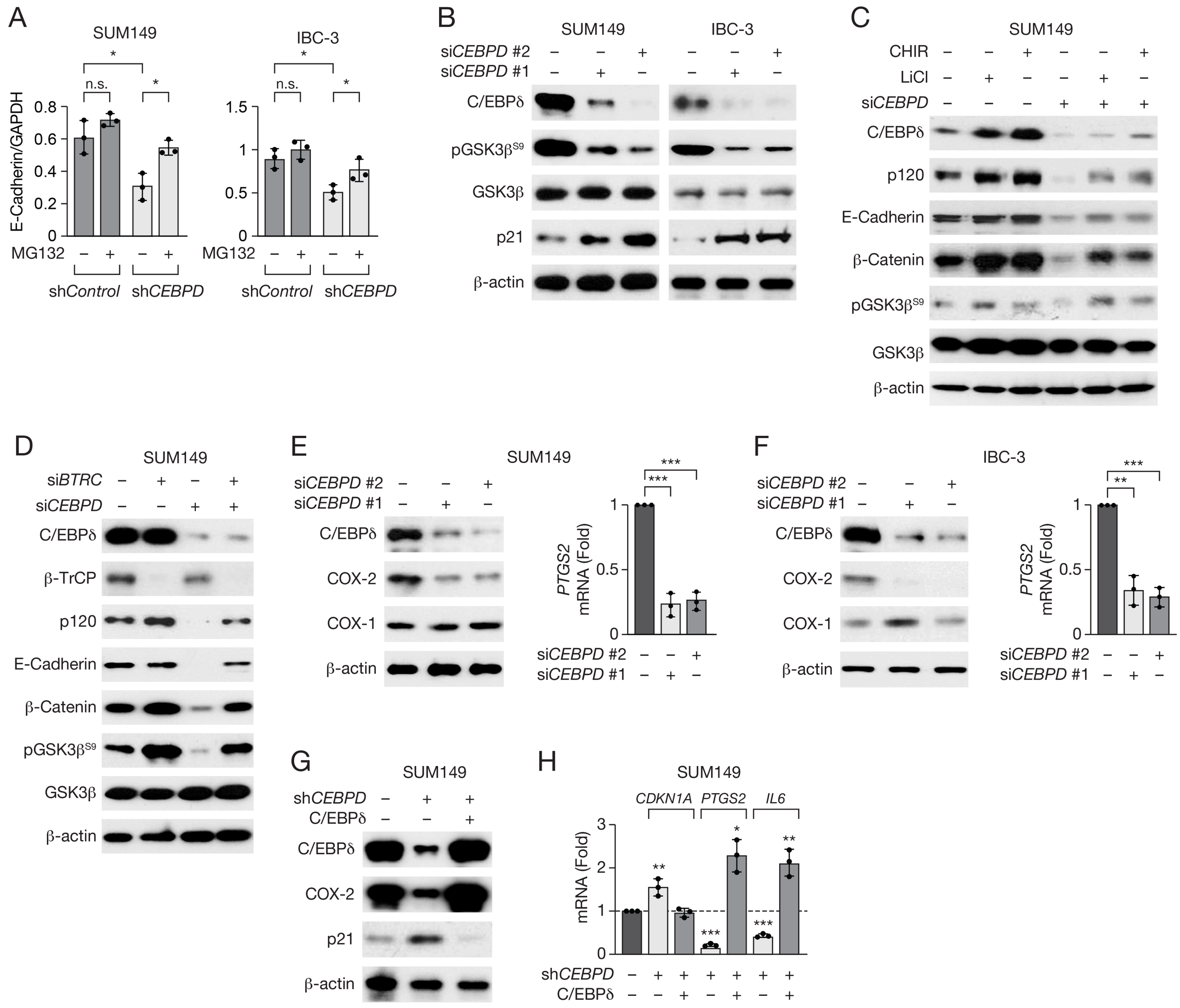

Supplemental Figure S2. C/EBPδ promotes E-cadherin protein stability through GSK3β inhibition and COX-2 gene transcription. (**A**) Quantification of E-Cadherin protein signal from Western analyses as in Figure 2A normalized to GAPDH loading control (n=3, mean ± SEM, **P*<0.05; n.s., not significant). (**B**) Western analysis of the indicated proteins in emboli from SUM149 and IBC-3 cells that were transfected with control or two independent si*CEBPD* oligos. (**C**) Western analysis of the indicated proteins in emboli from SUM149 cells transfected with control (-) or si*CEBPD* oligos and treated with LiCl (10 mM) or CHIR (5 µM) for 6 h. (**D**) Western analysis of the indicated proteins in emboli from SUM149 cells transfected with control (-) or si*CEBPD* along with si*BTRC* (β-TrCP) oligos. (**E-F**) Western (left) and mRNA analyses by qPCR (right) of COX-2 (*PTGS2*) expression in (E) SUM149 and (F) IBC-3 cells 72 h after transfection with si*Control* or two independent si*CEBPD* oligos (n=3, mean±SEM, ***P*<0.01, ****P*<0.001 compared to si*Control*). (**G**) Western analysis of the indicated proteins in SUM149 cells with stable expression of sh*Control* (-) or sh*CEBPD* (+) shRNA 72 h after transfection with vector (-) or C/EBPδ expression plasmid (+). (**H**) qPCR analysis of the indicated mRNA's in cells as in panel G (n=3, mean±SEM; **P*<0.05, ***P*<0.01, ****P*<0.001 compared to transfected with empty vectors).

Supplemental Figure S3

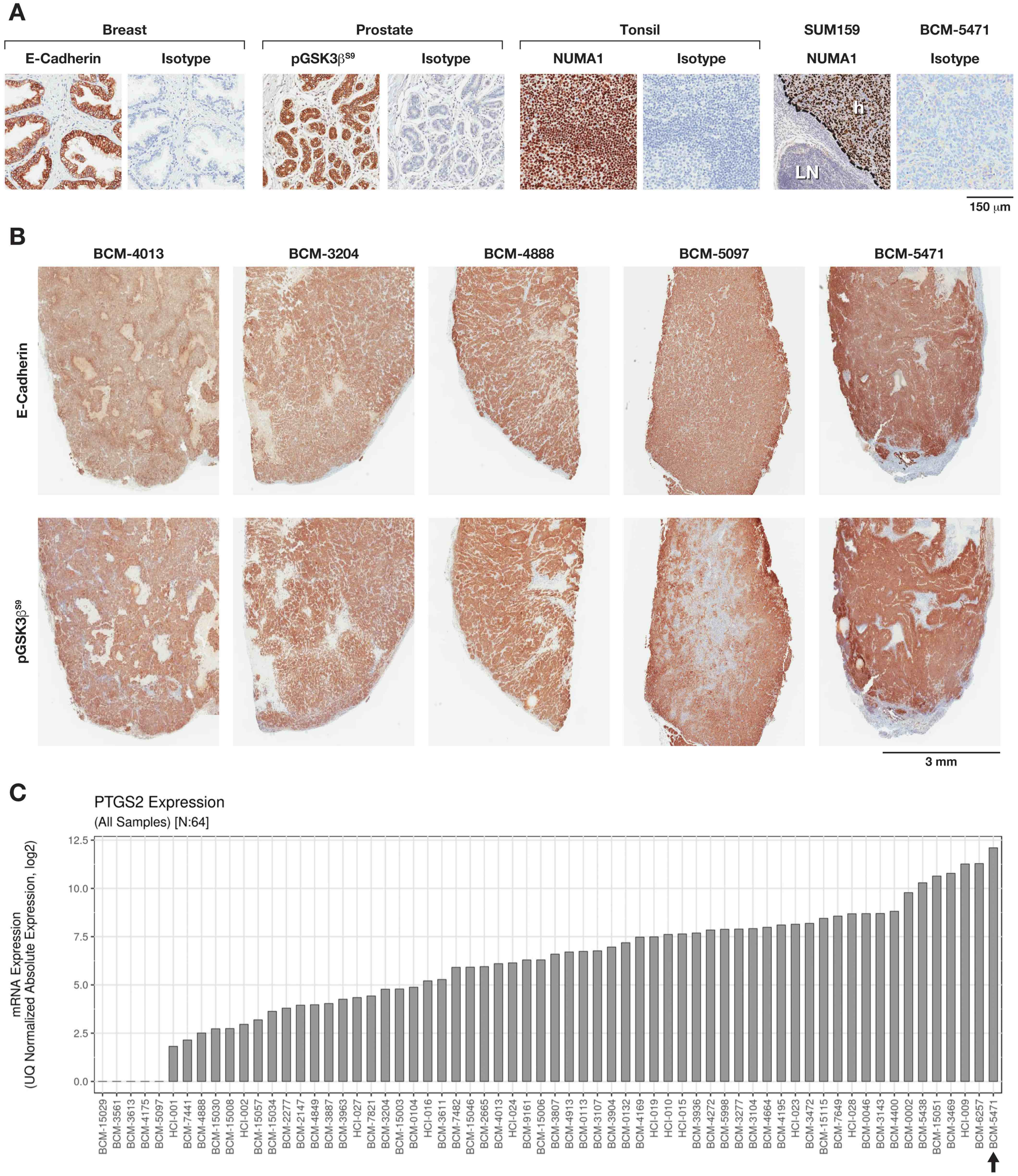

Supplemental Figure S3 continued

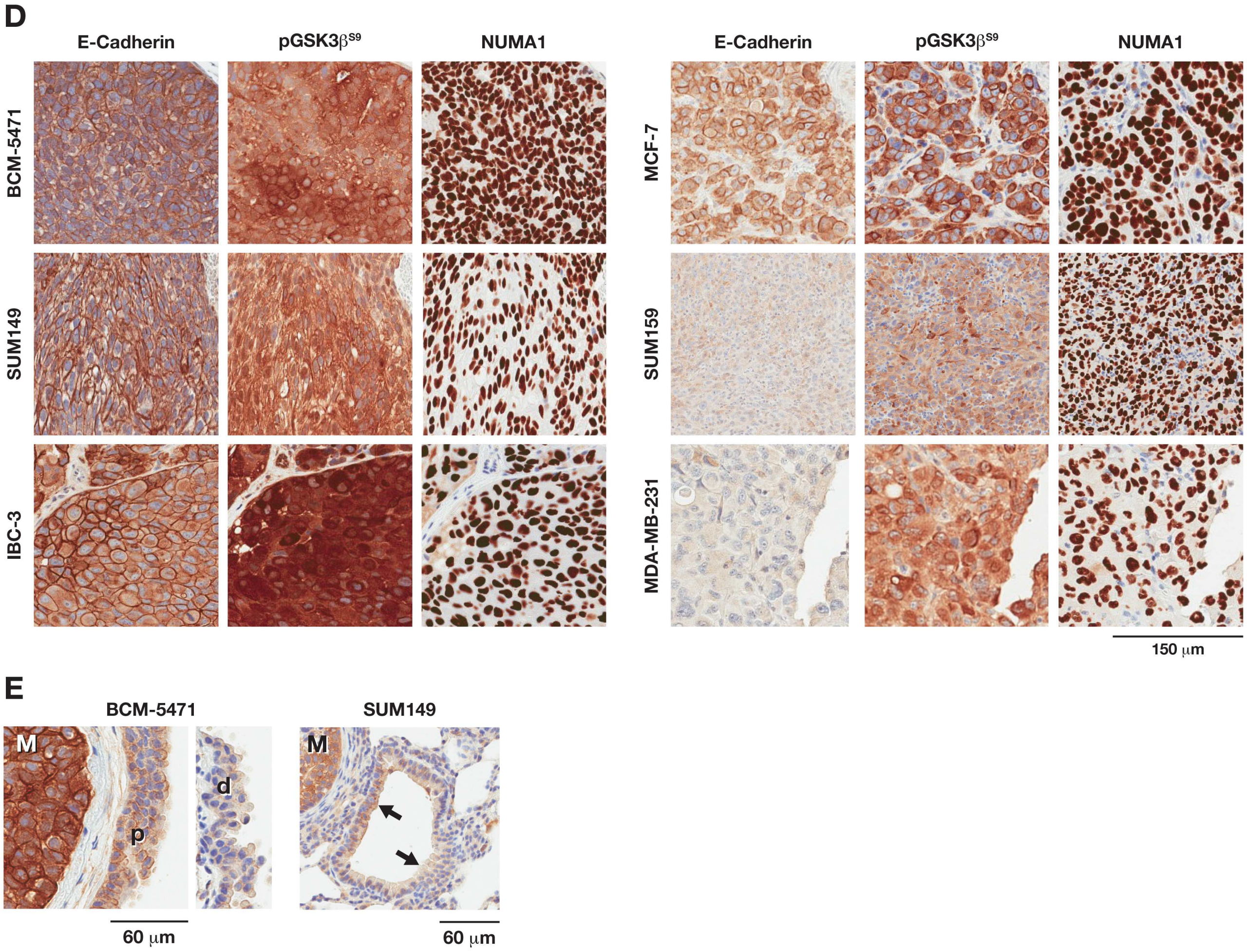

Supplemental Figure S3. Expression analysis of breast cancer xenograft primary tumors and lung metastases. (**A**) Antibody control samples showing immunostaining of E-Cadherin, pGSK3β^{S9} and human-specific NUMA1 on serial sections of normal human tissues as indicated and the respective isotype controls. NUMA1 staining of a SUM159 xenograft tumor denotes its specificity to human cells (h) while mouse stroma with lymph node (LN), demarcated by the dotted line, does not stain. A representative image of BCM-5471 tumor tissue shows lack of staining with isotype control antibodies. (**B**) Immunostaining of E-Cadherin and pGSK3β^{S9} on serial sections of primary tumors from the indicated PDX models, representing TNBC (BCM-3204, -4013, 5471), ER+/HER2+ (BCM-4888), and ER+ (BCM-5097) subtypes. (**C**) *PTGS2* mRNA data of BC PDX models as reported by the Patient-Derived Xenograft and Advanced In Vivo Models (PDX-AIM) Core at Baylor College of Medicine (https://pdxportal.research.bcm.edu). (**D**) Immunostaining of E-Cadherin, pGSK3β^{S9} and NUMA1 on primary tumor sections of PDX BCM-5471 and the indicated cell lines. (**E**) BCM-5471 and SUM149 lung metastases (M) with immunostaining of pGSK3β^{S9} (p/d, proximal/distal; black arrows indicate bronchial epithelium).

Supplemental Figure S4

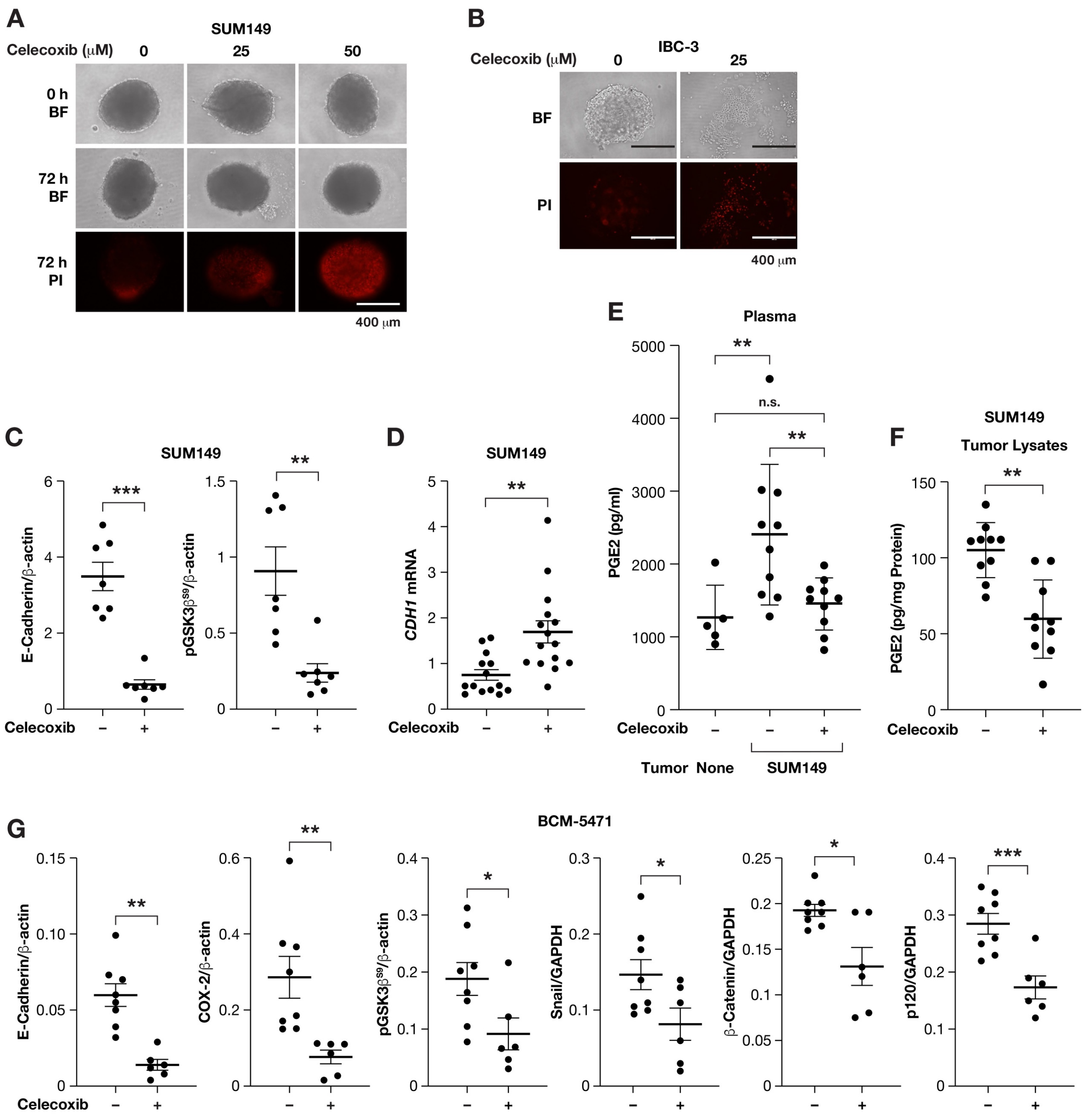

Supplemental Figure S4. Effect of celecoxib on established emboli in vitro and tumors in vivo. (**A**) Images of representative SUM149 cell emboli after 3 days of culture (0 h) and following another 72 h of treatment with celecoxib, as indicated, and stained with propidium iodide (PI, fluorescence imaging; BF, bright field). (**B**) Representative images of IBC-3 cells cultured in 3D ± celecoxib for 72 h and stained with PI (BF, bright field). (**C**) Quantification of E-Cadherin and pGSK3β^{S9} signals shown in Figure 4F of SUM149 tumors treated ± Celecoxib (n=7, mean±SEM; ***P*<0.01, ****P*<0.001 by unpaired two-sided Wilcoxon rank-sum test). (**D**) Quantification by qPCR analysis of *CDH1* mRNA in SUM149 tumors as in Figure 4F (n=14-15, ***P*<0.01 by unpaired Wilcoxon rank-sum test). (**E**) Concentration of PGE2 in plasma of mice bearing SUM149 tumors as shown in Figure 4F (n=10). Tumor-free mice (n=5) were used as control (mean±SEM; n.s., not significant, *P* values by unpaired two-sided Wilcoxon rank-sum test). (**F**) Quantification of PGE2 in SUM149 tumor tissue extracts from mice as in Figure 4F (n=10, mean±SEM; n.s., not significant; ***P*<0.01 by unpaired two-sided Wilcoxon rank-sum test). (**G**) Quantification of Western analysis shown in Figure 4I of BCM-5471 PDX tumors treated ± Celecoxib (n=6-8, mean±SEM; **P*<0.05, ***P*<0.01, ****P*<0.001 by unpaired two-sided Wilcoxon rank-sum test).

Supplemental Figure S5

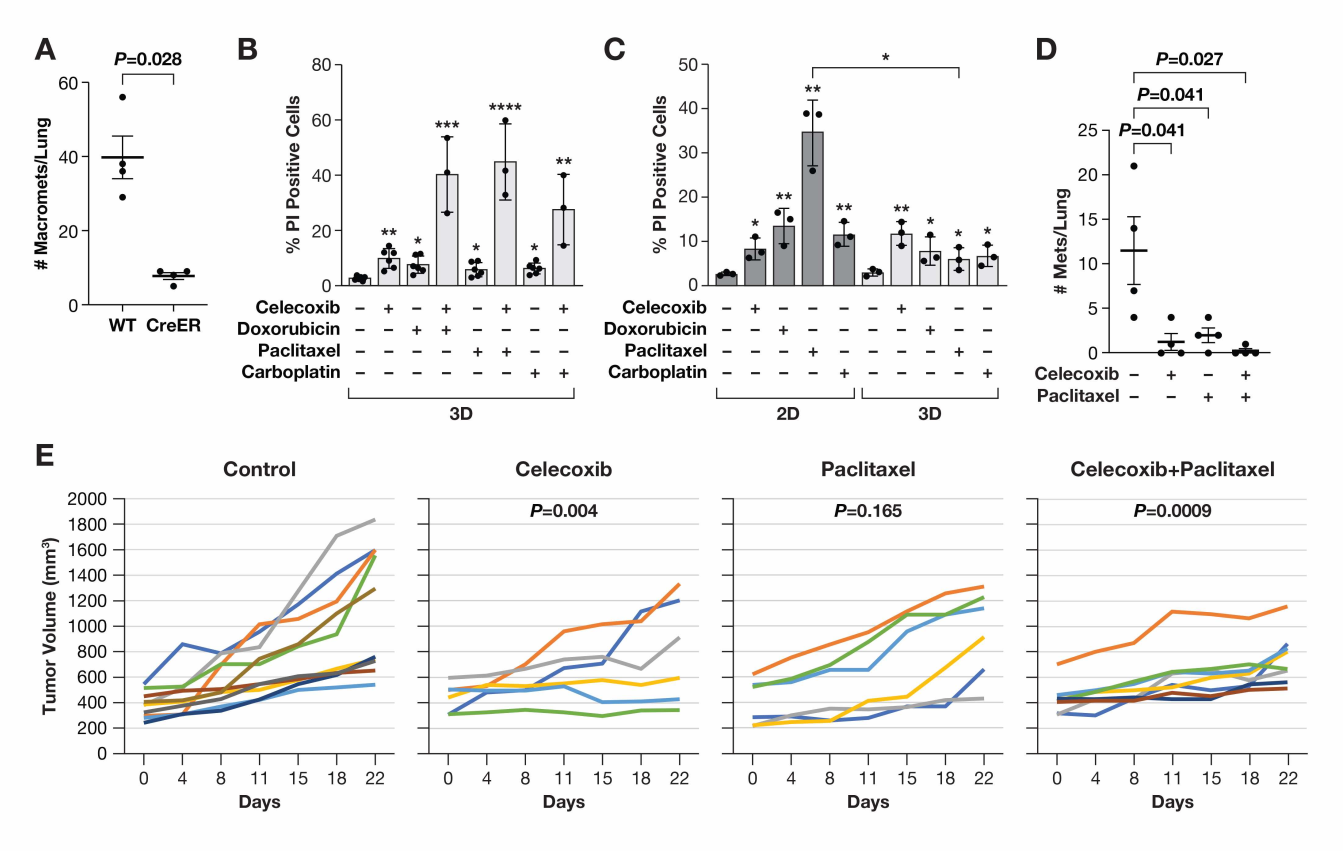

Supplemental Figure S5. Effect of *Cdh1* deletion in seeded metastases and assessment of celecoxib and paclitaxel drug sensitivity in vitro and in vivo. (**A**) Quantification of macrometastases in lungs of mice injected with E-cadfl/fl or E-cadfl/fl; CreER expressing cells, followed 1 week later by tamoxifen injection to delete the E-cadherin gene, and harvested 3 weeks thereafter (n=4, *P* value by two-tailed non-parametric t-test, Mann Whitney). (**B**) Quantification of propidium iodide (PI) positive cells in emboli treated after 3 days of 3D culture for additional 3 days with individual drugs (50 µM celecoxib, 500 nM doxorubicin, 50 nM paclitaxel, 10 µM carboplatin) and/or combinations as indicated (n=3, mean±S.E.M.; **P*<0.05, ***P*<0.01, ****P*<0.001, *****P*<0.0001 compared to DMSO control). (**C**) Quantification of PI positive cells from cells cultured in 2D or 3D and treated as in panel B (n=3, mean±S.E.M.; **P*<0.05, ***P*<0.01 compared to DMSO control or as indicated by bracket). (**D**) Quantification of tumor cell colonies (mets) in lungs of mice injected with SUM149 cells and treated with celecoxib (1000 mg/kg chow) and/or paclitaxel (10 mg/kg i.v.), n=4, P as indicated by two-sided unpaired Wilcoxon test compared to untreated. (**E**) Tumor volume measurements of BCM-5471 PDX's in mice treated with celecoxib (1000 mg/kg chow) and/or paclitaxel (10 mg/kg i.v.), n=6-10, *P* values were determined by a linear model of treatment by day interaction relative to control baseline.

Supplemental Figure S6

NM_005195.3 CEBPD mRNA

```
AGGTGACAGCCTCGCTTGGACGCAGAGCCCGGCCCGACGCCGCCATGAGCGCCGCGCTCTTCAGCCTGGA   70
CGGCCCGGCGCGCGGCGCGCCCTGGCCTGCGGAGCCTGCGCCCTTCTACGAACCGGGCCGGGCGGGCAAG   140
CCGGGCCGCGGGGCCGAGCCAGGGGCCCTAGGCGAGCCAGGCGCCGCCGCCCCCGCCATGTACGACGACG   210
AGAGCGCCATCGACTTCAGCGCCTACATCGACTCCATGGCCGCCGTGCCCACCCTGGAGCTGTGCCACGA   280
CGAGCTCTTCGCCGACCTCTTCAACAGCAATCACAAGGCGGGCGGCGCGGGGCCCCTGGAGCTTCTTCCC   350
          TCGCCGACCTCTTCAACAG=siRNA#1

GGCGGCCCCGCGCGCCCCTTGGGCCCGGGCCCTGCCGCTCCCCGCCTGCTCAAGCGCGAGCCCGACTGGG   420
GCGACGGCGACGCGCCCGGCTCGCTGTTGCCCGCGCAGGTGGCCGCGTGCGCACAGACCGTGGTGAGCTT   490
GGCGGCCGCAGGGCAGCCCACCCCGCCCACGTCGCCGGAGCCGCCGCGCAGCAGCCCCAGGCAGACCCCC   560
GCGCCCGGCCCCGCCCGGGAGAAGAGCGCCGGCAAGAGGGGCCCGGACCGCGGCAGCCCCGAGTACCGGC   630
AGCGGCGCGAGCGCAACAACATCGCCGTGCGCAAGAGCCGCGACAAGGCCAAGCGGCGCAACCAGGAGAT   700
                                                     pDEST_SIGMA=AACCAGGAGAT

GCAGCAGAAGTTGGTGGAGCTGTCGGCTGAGAACGAGAAGCTGCACCAGCGCGTGGAGCAGCTCACGCGG   770
GCAGCAGAAGT
GACCTGGCCGGCCTCCGGCAGTTCTTCAAGCAGCTGCCCAGCCCGCCCTTCCTGCCGGCCGCCGGGACAG   840
CAGACTGCCGGTAACGCGCGGCCGGGGCGGGAGAGACTCAGCAACGACCCATACCTCAGACCCGACGGCC   910
CGGAGCGGAGCGCGCCCTGCCCTGGCGCAGCCAGAGCCGCCGGGTGCCCGCTGCAGTTTCTTGGGACATA   980
GGAGCGCAAAGAAGCTACAGCCTGGACTTACCACCACTAAACTGCGAGAGAAGCTAAACGTGTTTATTTT   1050
                               siRNA#2=CCACTAAACTGCGAGAGAA
                                    (Dox)SMARTchoice=CGAGAGAAGCTAAACGTGTTTATTTT

CCCTTAAATTATTTTTGTAATGGTAGCTTTTTCTACATCTTACTCCTGTTGATGCAGCTAAGGTACATTT   1120
GTAAAAAGAAAAAAAACCAGACTTTTCAGACAAACCCTTTGTATTGTAGATAAGAGGAAAAGACTGAGCA   1190
TGCTCACTTTTTTATATTAATTTTTACAGTATTTGTAAGAATAAAGCAGCATTTGAAATCGAAAAAAAAA   1260
```

Supplemental Figure S6. ***CEBPD* targeting sequences.** The human *CEBPD* sequence (NM_005195.3) is shown along with the aligned sequences of each siRNA and shRNA (pDEST_SIGMA) used in this study. The translation start and stop codons are indicated by shaded boxes.
